# Design Optimization for Rough Terrain Traversal Using a Compliant, Continuum-Joint, Quadruped Robot

**DOI:** 10.3389/frobt.2022.860020

**Published:** 2022-07-11

**Authors:** Vallan Sherrod, Curtis C. Johnson, Marc D. Killpack

**Affiliations:** Robotics and Dynamics Laboratory, Department of Mechanical Engineering, Brigham Young University, Provo, UT, United States

**Keywords:** multi-objective optimization, design metrics, genetic algorithm, evolutionary optimization, quadruped design, continuum robot, soft robot, configuration space approximation

## Abstract

Legged robots have the potential to cover terrain not accessible to wheel-based robots and vehicles. This makes them better suited to perform tasks such as search and rescue in real-world unstructured environments. In addition, pneumatically-actuated, compliant robots may be more suited than their rigid counterparts to real-world unstructured environments with humans where unintentional contact or impact may occur. In this work, we define design metrics for legged robots that evaluate their ability to traverse unstructured terrain, carry payloads, find stable footholds, and move in desired directions. These metrics are demonstrated and validated in a multi-objective design optimization of 10 variables for a 16 degree of freedom, pneumatically actuated, continuum joint quadruped. We also present and validate approximations to preserve numerical tractability for any similar high degree of freedom optimization problem. Finally, we show that the design trends uncovered by our optimization hold in two hardware experiments using robot legs with continuum joints that are built based on the optimization results.

## 1 Introduction

Animals and legged robots are generally able to traverse more terrain than wheeled or tracked vehicles. Therefore we expect that the use of a legged robot as opposed to a wheeled robot would greatly increase the regions where the robot can operate. Legged robots are especially capable of performing tasks in unstructured environments alongside humans instead of being limited to factories or other highly structured environments. However, operating safely around humans in unstructured environments is difficult for traditional rigid, position-controlled robots.

Traditional rigid position-controlled robots present dangers to humans because of their high reflected inertia due to their high gear ratios. These reflected inertias cause high contact forces when colliding with objects present in unstructured environments that can be damaging to both the robot and the objects. While force control can be a solution to this problem for rigid robots, force control is inherently difficult for stability reasons. The result is that there has been a recent trend in exploring robot designs made from softer materials and powered by soft actuation to allow for more passive compliance as opposed to traditional rigid position-controlled robots. These kinds of robots are known as soft robots. These soft robots are safer to operate around humans and in unstructured environments because incidental contact forces can be greatly reduced and high contact forces can be mitigated with the natural compliance of the platform.

Soft continuum-joint robotics legs in conjunction with soft manipulators can provide the groundwork to perform whole-body, dynamic, mobile manipulation in soft robotics. Work in mobile manipulation is relatively new for any kind of quadruped robot. Boston Dynamics (a commercial enterprise) with their SpotMini is one of the only groups to have demonstrated control of a quadruped with an arm attached. Spot is a rigid robot with compliance introduced through impedance control. However, if the whole system was compliant by nature, the platform would be safer around humans and less likely to damage itself when it falls. In addition, robots with compliant legs have the possibility of adapting to rough terrain more effectively (Godage et al. (2012)) than their rigid counterparts.

The research in this paper is focused on methods for designing such a compliant platform. Specifically, our goal is to develop and validate useful metrics to aid in the design optimization of a 16-degree-of-freedom (DoF), pneumatically-actuated, continuum-joint, soft quadruped robot whose goal applications are mobility in unstructured environments and mobile manipulation. Our general contributions are two-fold: 1) we introduce a general methodology for the design optimization of a large-scale quadruped and 2) we provide important simplifications and approximations to maintain computational tractability. While this paper specifically focuses on a continuum-joint soft quadruped, these contributions are applicable to other quadruped platforms–rigid, soft, hybrid, or otherwise.

## 2 Related Work

The validation test bed presented in this paper is not entirely soft like many of the platforms used for soft robot locomotion, nor is it entirely rigid like many of the platforms used for legged robot design metrics and optimization. Hence this section summarizes work done in each field in order to provide context for the work in this paper which is inherently a mixture between both.

### 2.1 Legged Robot Design Metrics and Optimization

Little research exists regarding metrics for a design optimization of a legged, soft robot. There is design optimization research for traditional rigid, legged robots such as the genetic algorithms used in Birglen and Ruella (2014), Fedorov and Birglen (2015), and Gülhan and Erbatur (2018). However, most of the research considers designs with fewer than ten design variables which are not directly applicable to a legged, soft robot. Because the design metrics formed for these optimizations are not sufficient to aid directly in this work, we have developed new design metrics tailored specifically to a legged soft robot.

Chadwick et al. (2020) recently contributed an open source design optimization toolbox that uses evolutionary algorithms and allows user-specific metrics, The paper itself, however, provides little guidance as to what metrics to use or what design trade-offs exist. In Uno et al. (2021), the authors introduce metrics that are similar in nature to ours (i.e. attempting to measure the ability to traverse rough terrain), but they do not use the metrics to produce novel designs or explore trade offs in the metrics as shown in this paper. Semasinghe et al. (2021) is one of the most relevant papers which introduces three design metrics for the design optimization of the quadruped robot: the system energy consumption, the passive impedance torque, and the stepping time. We build on this work by presenting metrics that also treat stability, payload, and desired velocity capabilities which are important factors for applications such as search and rescue. We also explore tradeoffs in the Pareto front and provide hardware validation of the optimization results using a physical prototype, built using several optimized designs.

Other notable work includes Roennau et al.’s optimization of the leg mounting configuration on their stick insect inspired robot (LAURON V) by attempting to maximize a manipulability measure along several planes in the workspace Roennau et al. (2014). De Vincenti et al. (2021) and Ha et al. (2018) optimized both design and control of quadrupedal robots simultaneously. Both approaches used relatively few design parameters (i.e. less than 10) due to using gradient-based optimizers. Additionally, there is little treatment of metrics that are important for rough terrain traversal. Our paper contributes several intuitive metrics that are adapted for rough terrain.

### 2.2 Soft-Robot Locomotion

Land-based soft robot locomotion research is still rather nascent. The most promising and relevant of the previous work (in terms of payload) is research from Godage et al., in 2012. They built a small quadruped robot using pneumatically-actuated continuum limbs Godage et al. (2012). Each of these limbs has two degrees of freedom. They demonstrate remarkable capabilities including open-loop walking on flat and uneven terrain Godage et al. (2012). Shepherd et al. also achieve similar open-loop locomotion success with a small soft robot made primarily from elastomeric polymers Shepherd et al. (2011). Many researchers have produced similar designs looking at open-loop locomotion for lightweight soft continuum crawling robots (see Onal and Rus (2013); Florez et al. (2014); Wang et al. (2014); Rogóż et al. (2016); Vikas et al. (2016); Cao et al. (2018); Zou et al. (2018); Qin et al. (2019), and a survey on soft crawling robots in Chen et al. (2020)). However, as far as we can tell, none of these platforms were designed with specific locomotion metrics in mind. Instead, they tended to optimize the gait after the robot was already built.

There has been some work on multi-legged soft or compliant robots [see Zhang et al. (2021); Kim et al. (2021)]. Perhaps the paper most similar to our work uses an evolutionary algorithm to optimize the shape of a soft robot leg [see Morzadec et al. (2019)]. However, they do not describe how this approach might scale as the number of degrees of actuated degrees of freedom, the number of legs, and the number of desired design metrics increase. Instead, their approach is presented more generally than how to design compliant continuum joint robot for locomotion. Because the platform designed in this work is based on optimizing specific metrics and examining the trade-offs between them, we expect that it should be capable of much larger payloads and higher speeds than previous work in related untethered soft robot locomotion. We have also added closed-loop feedback configuration control of the limbs in order to evaluate force output at different locations in the leg’s workspace.

Most of the other work on soft-robot locomotion involves bioinspired platforms such as starfish Jin et al. (2016), salamanders Crespi et al. (2013), and octopi Cianchetti et al. (2015). A majority of these platforms are small scale–with most being under 30 cm long (the longest is a meter in length, but only 6 cm tall). Because of their small scale these platforms are incapable of lifting heavy payloads and therefore unsuitable for applications that require mobility while carrying a significant payload (e.g., search and rescue). Our goal is to develop, optimize, and build a platform suitable for such use cases in this work, while still building on the apparent strengths of soft continuum robot joints for locomotion in unstructured terrain.

## 3 Metric Definitions and Approximations

In this section, we introduce the four general metrics for the optimization of a quadruped robot with soft continuum joints and also discuss several approximations of these metrics that preserve numerical tractability. The metrics are formulated to capture various aspects of performance and control of a quadruped design but their use is not limited to four-legged designs. The design metrics and their corresponding approximations are developed in-depth in each subsequent section and are listed here for convenience:

1. Dexterity in Walking Regions (Section 3.1)
2. Average Payload in Walking Regions (Section 3.2)
3. Average Static Stability (Section 3.4)
4. Average Desired Velocity (Section 3.5)

The design metrics 1 and 2 are fairly straightforward and can be calculated directly. However metrics 3 and 4 are part of a class of metrics which, based on our optimization method, require an evaluation across the entire robot workspace which would not nominally be tractable. For this reason, we first present metrics 1 and 2. Then we present a generalization of how to calculate metrics 3 and 4 which can apply to any other similar metric, before we present the specifics of the static stability and average desired velocity metrics.

**FIGURE 1:**
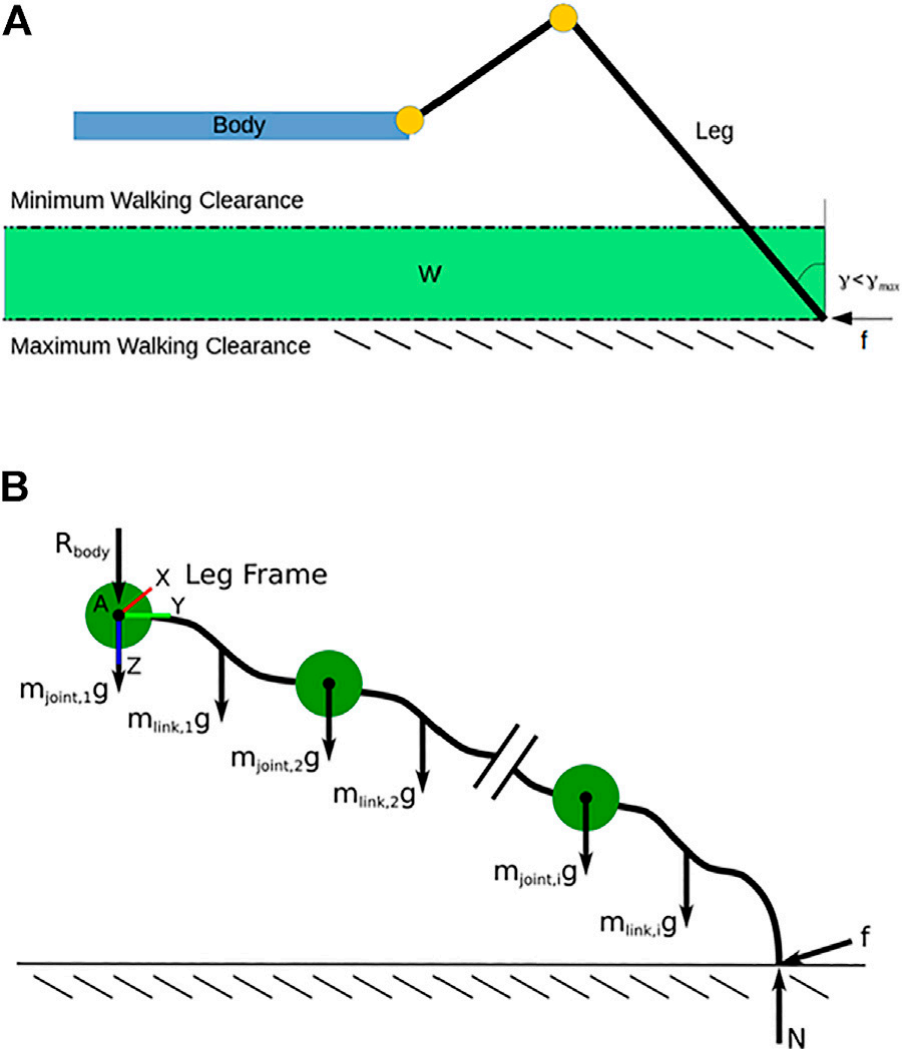
**(A)** A visualization of the walking region *W*. **(B)** The free body diagram of a single leg cut away from the body and making contact with the ground. The green circles represent joints as lumped particles, the black lines represent rigid links whose actual geometry is being optimized, and the dotted line represents the ground plane.

For the calculation of each metric, we define the possible terrain a robot may face in real-world situations as the walking region (*W*) which consists of all the 3D Cartesian points located in an infinite volume bounded by the minimum and maximum walking clearance from the bottom of the robot (visualized in Figure 1A). The minimum and maximum walking clearances correspond to a minimum and maximum desired walking height for the body of the robot over a given terrain. This taskspace is six-dimensional as it combines the 3D position of the foot in *W* with its corresponding 3D orientation vector (i.e., the pose of the foot).

In order to efficiently sample from this taskspace, we use the discretization presented by Bodily (2017)–though any discretization can be used. This consists of discretizing the taskspace into a *M* ^3^ × *N* ^3^ rectangular grid. *M* ^3^ is the 3D tensor that represents the discretization of the foot position in Cartesian Space. *N* ^3^ is the 3D tensor for the discretization of the foot orientation (represented as an axis-angle vector) at each discrete Cartesian position of *M* ^3^. A given foot pose is assigned to a bin in this discretization by finding the nearest Cartesian discretization point to its position in *M* ^3^ and subsequently locating the nearest orientation discretization point in the *N* ^3^ grid associated with this Cartesian point.

### 3.1 Dexterity in Walking Regions

#### 3.1.1 Metric Definition

The dexterity in walking region metric represents the number of foothold combinations a particular quadruped design can reach during online control and planning. The point in optimizing this metric is that if more footholds are found, then 3D rough terrain planners such as in Loc et al. (2010) or Zhang et al. (2019) should find better solutions.

We find how many footholds a leg can reach in the discretized taskspace *W* by sampling the leg’s configuration space. We do this by sweeping each of the leg’s joints from their minimum to maximum values at a set resolution recursively. At each configuration, we check if the foot is located in *W* using forward kinematics. If the foot pose is in *W* and is within an orientation tolerance of *γ*_max_ from the vertical (see Figure 1A), we mark the corresponding pose “bin” of *W* as reachable. The orientation tolerance eliminates unrealistic poses that cannot result in suitable footholds (e.g., the foot facing upward or parallel with the ground). The choice of *γ*_max_ is dependent on the foot design because it is related to the coefficient of friction, *μ*, between the foot and the ground. For a given *μ*, there is a critical angle at which the foot will slide based on a static analysis of the foothold. Consequently, *γ*_max_ must be less than this critical angle. This implies that foot designs with higher *μ* values allow for greater values of *γ*_max_. The *dexterity in walking region* metric for a single leg, *n*_*W*,*i*_, is the number of unique pose bins in *W* that are reachable.

To calculate the *dexterity in walking region* metric for the full legged robot, *n*_foot_, we consider all reachable foothold combinations by multiplying all the *n*_*W*,*i*_ together: $n_{W,\text{all}}=\prod_{i=1}^{n_{\text{leg}}}n_{W,i}$ , where *n*_leg_ is the number of legs on the full legged robot. For example, consider a 5 legged robot (i.e. where *n*_leg_ = 5) where the first, second, third, fourth, and fifth legs have 3, 4, 5, 6, and 7 reachable footholds respectively. This means that *n*_*W*,all_ = (3 × 4 × 5 × 6 × 7) = 2520 combinations for this robot.

Note that *n*_*W*,all_ only includes combinations where all the feet are in *W*. Static stability (which is discussed in more detail in Section 3.4) only requires that three legs be on the ground at all times. To find all possible combinations where at least three legs are in the walking region (i.e., *n*_foot_), we use

$$n_{\text{foot}}=n_{W,all}\overset{n_{\text{leg}}}{\underset{r=3}{\sum }}C\left(n_{\text{leg}},r\right)$$ (1)

where *C*(*n*_leg_, *r*) is the number of combinations of *r* legs being chosen from the total number of legs, *n*_leg_:

$$C\left(n_{\text{leg}},r\right)=\frac{n_{\text{leg}}!}{\left(n_{\text{leg}}-r\right)!r!}$$ (2)

#### 3.1.2 Metric Approximation

For our optimization, we simplify this metric in two ways. First, since the optimization is limited to quadrupeds, the summation in Eq. 1 simplifies to a constant 5 (*n*_leg_ = 4 ⇒ *C*(4, 3) + *C*(4, 4) = 4 + 1). Second, because we require leg designs to be symmetric we only need to evaluate the *dexterity in walking region* metric for one leg as *n*_*W*,1_ = *n*_*W*,2_ = *n*_*W*,3_ = *n*_*W*,4_ in this case. Substituting these simplifications into Eq. 1 reduces the expression to $n_{\text{foot}}=5n_{W,1}^{4}$ . Since this is a strictly increasing function for *n*_*W*,1_ > 0, maximizing *n*_*W*,1_ is the same as maximizing *n*_foot_. Therefore, we simply use *n*_*W*,1_ in the optimization.

### 3.2 Average Payload in Walking Regions

#### 3.2.1 Metric Definition

This metric quantifies a legged robot’s ability to statically support a payload during operation. We choose to do the analysis statically because the robot is designed to operate under a static gate. The premise of the *average payload in walking regions* metric is as follows.

For each combination where at least three leg configurations exist in *W* (i.e., one of the combinations of leg configurations that counted for *n*_foot_ as described in Section 3.1) we calculate the required joint efforts (*τ*) to support the weight of the robot (*F*_robot_). We ensure that each *τ* satisfies

$$\tau_{\text{min}}\leq \tau \leq \tau_{\text{max}}$$ (3)

where *τ*_min_ and *τ*_max_ are joint torque limits. If each joint torque in *τ* is not within the limits, we assign a payload capability (*F*_payload_) of zero to the configuration. However if they are all within the limits, we calculate the maximum weight that the robot design can theoretically support (*F*_max_) by saturating the joint whose effort is closest to its limit and estimating the resulting force output. *F*_payload_ is then calculated as

$$F_{\text{payload}}=F_{\text{max}}-F_{\text{robot}}.$$ (4)

Some configurations that are close to singularities allow *F*_max_ to approach infinity because these configurations are limited by the strength of the structure of the robot as opposed to joint-effort limits in their load bearing capacity. Therefore, we compute the payload score for a specific configuration (*S*_payload_) as *S*_payload_ = min(*F*_payload_, *F*_payload,_ _max_) where *F*_payload,_ _max_ is chosen based on the robot structure (i.e. a force that is lower than a critical buckling, axial, or shear force on the structure). The *average payload in walking region* metric is then computed as the average of all the *S*_payload_ over all the valid configurations.

#### 3.2.2 Metric Approximation

In our case, the symmetry of the quadruped design allows us to approximate the *average payload in walking region* metric by finding *F*_payload_ for a single leg. This is valid if we use a good estimate for the load a single leg needs to support relative to the total weight of the robot. Accordingly, this section derives a model used to determine the joint efforts required from a single leg given an estimate of *F*_robot_. This section also demonstrates how to find *F*_max_ with the joint effort which is closest to its limit.

Figure 1B shows a FBD of a single leg cut away from the base of the robot. Point A is where the shoulder of the leg connects with the body of the robot. *R*_body_ is the reaction force from the body at Point A. Summing moments about A gives

$$\tau =\overset{n}{\underset{i=1}{\sum }}\left(J_{\text{link,}i}^{T}m_{\text{link,}i}g+J_{\text{joint,}i}^{T}m_{\text{joint,}i}g\right)+J_{\text{foot}}^{T}N+J_{\text{foot}}^{T}f,$$ (5)

where *n* is the number of links and $J_{X,i}\in R^{3\times \left(\text{\# of joints}\right)}$ refers to the manipulator Jacobian relating the Cartesian velocities of point *X* (i.e., center of gravity of a link or a joint) in the leg base frame (the coordinate frame in Figure 1B) to the joint velocities.

In walking, friction *f* is only present when the leg attempts to accelerate by pushing against the ground. This is either to push the leg forward before lift-off or as it pushes back as the leg makes contact after a swing. We restrict the calculations for the *average payload in walking region* metric to cases where the friction is zero since a legged robot may have multiple desired walking directions. The zero-friction case corresponds to the robot being able to support itself while standing still or in the middle of a gait and not applying a force along the plane of the ground to accelerate. The *average desired velocity metric*, presented in Section 3.5, will investigate the robot’s ability to provide a force in a desired walking direction. In these cases, we assume that the robot is able to apply a force only in the normal direction without applying any horizontal forces along the ground that would produce a friction force. With friction, *f*, being zero, Eq. 5 simplifies to

$$\tau =\overset{n}{\underset{i=1}{\sum }}\left(J_{\text{link,}i}^{T}m_{\text{link,}i}g+J_{\text{joint,}i}^{T}m_{\text{joint,}i}g\right)+J_{\text{foot}}^{T}N,$$ (6)

where the only unknown for a given configuration is the normal force, *N*.

**FIGURE 2:**
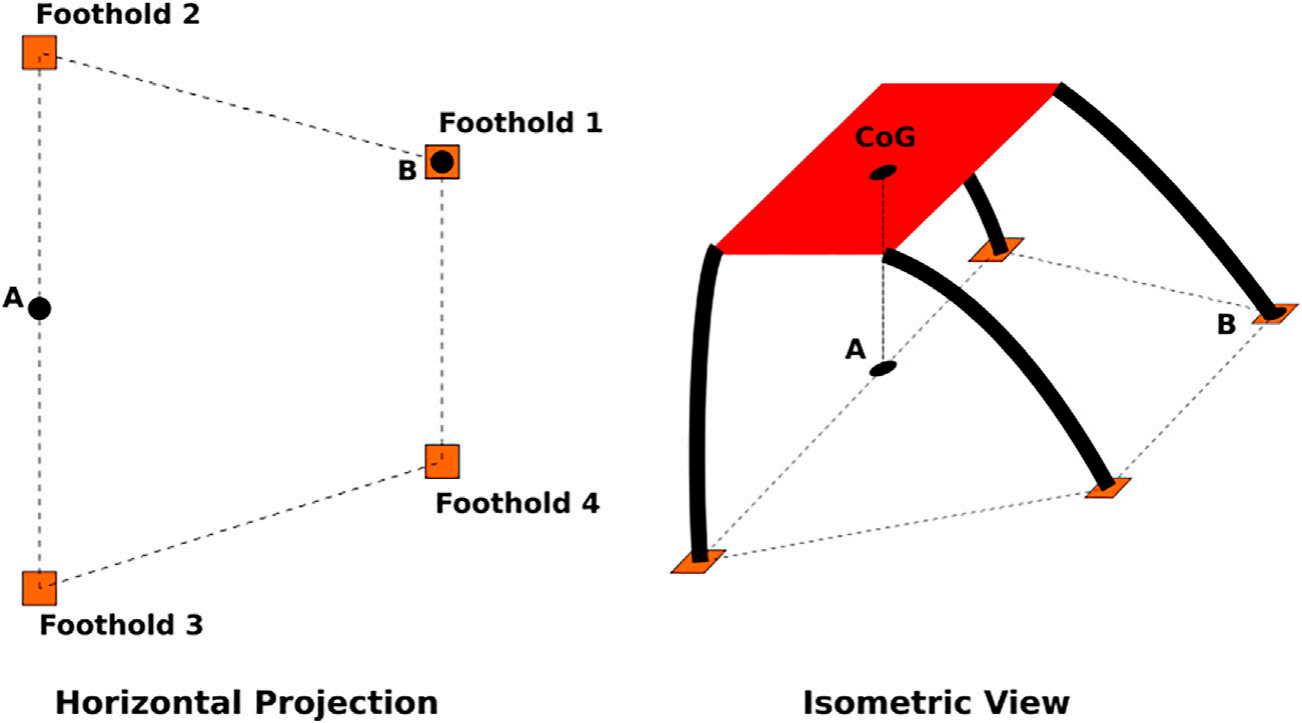
Diagram showing the horizontal foothold projection (left) and an simplified isometric view of the robot (right). Static analysis shows the force required of the leg at foothold 1 has a lower bound of *N* = 0 occurs when the CoM is over point A, as shown in the isometric view. The upper bound *N* = *F*_*robot*_/2 occurs when the CoM is over point B.

The value of *N* for a single leg is dependent on the location of the center of gravity of the robot and the normal forces from all the other legs. We choose an approximate value for *N* that reasonably represents the average load the leg is required to bear since we are only considering one leg and cannot explicitly calculate it. Figure 2 illustrates the possible values of *N* depending on the location of the center of gravity of the robot with respect to the location of the footholds of the other legs. Without loss of generality for other footholds, we restrict our analysis to the leg at foothold 1. A static force balance shows that the lower bound for *N* is zero when the center of gravity is located above point A. The upper bound of *N* (when the robot is static) is the full weight of the robot when the center of gravity of the robot is directly over point B. All other locations of the center of gravity of the robot (in static equilibrium) result in a value of *N* between these bounds. Therefore, a reasonable choice for *N* is half the weight of the robot (i.e., $N=\frac{F_{robot}}{2}$ ). While this may seem a bit ambiguous, we see later in this section why the specific choice of *N* does not greatly affect the trends we desire to discover from this metric in optimization problems.

With this choice for *N*, we can calculate the joint efforts required to support *N* using Eq. 6. As mentioned in Section 3.2.1, if *τ* satisfies Eq. 3 we approximate *F*_max_ where at least one of the joint efforts is saturated and the other joint efforts are not. In Eq. 6 the summation term represents the joint efforts required to resist gravity. We will refer to this term as *τ*_g_. We desire to scale *N* by a scalar, *s*, such that at least one joint effort *τ*_*i*_ ∈ *τ* is at its limit and the rest remain within their joint-effort limits. Thus we have $\tau =\tau_{g}+J_{\text{foot}}^{\text{T}}sN$ , where *τ* satisfies Eq. 3 with at least one of its elements equal to either its corresponding *τ*_min_ or *τ*_max_. Since *s* is a scalar, this equation can be rewritten as

$$\tau =\tau_{\text{g}}+sJ_{\text{foot}}^{\text{T}}N.$$ (7)

The value of *s* is the minimum value required to saturate *τ*_*i*_ while all other elements of *τ* satisfy Eq. 3:

$$s=\text{min}\left(\frac{\tau_{\text{max/min, }i}-\tau_{\text{g,}i}}{\tau_{N,i}}\right)$$ (8)

where $\tau_{N}=J_{\text{foot}}^{T}N$ . Here, *τ*_max/min,__*i*_ is either the maximum or minimum joint effort a joint may have in its given configuration as described by its joint model. Whether the maximum or minimum joint effort is used is determined by the sign of *τ*_*N*,*i*_. If *τ*_*N*,*i*_ is positive, we use the maximum, and if *τ*_*N*,*i*_ is negative, we use the minimum. This is because the scaling of *N* only causes a change in joint efforts in the direction of *τ*_*N*,*i*_. *F*_max_ can then be approximated with *s* as *F*_max_ ≈ *sN*. Therefore, the approximation of the payload capability in Eq. 4 becomes

$$F_{\text{payload}}\approx sN-N=\frac{F_{robot}}{2}\left(s-1\right).$$ (9)

This tells us how much additional force can be applied beyond the nominal *N* needed to support the robot weight. This approximation is then saturated with the *F*_payload,_ _max_ to arrive at the full, approximated metric, ${\hat{S}}_{\text{payload}}$ . Recall that ${\hat{S}}_{\text{payload}}$ represents only the payload capabilities of a single leg. However, when all the legs are symmetric, it can be seen that maximizing this approximation will be the same as maximizing the robot’s overall payload capabilities, *S*_payload_.

We can evaluate our choice of *N* as half the weight of the robot using Eq. 9. *N* changes based on the location of the center of gravity which results in different values of *s* at each robot configuration. However, the relationship *F*_max_ ≈ *sN* remains the same since this is an actual physical limit of how much force a given configuration can support. A higher value of *N* increases the number of configurations where Eq. 3 does not hold and will not contribute to the payload metric because they would be zeroed out. A lower value has the opposite effect. However, trends for higher-payload robots are still distinguishable in an optimization regardless of the choice of *N* since the only information loss occurs with low-payload designs that are zeroed out. Therefore, the specific choice of *N* has minimal effect on being able to find robots with overall higher payload capabilities.

### 3.3 General Legged Robot Configuration Space Approximation

Recall that all of the design metrics presented in this paper require calculations over the entire configuration space of a legged robot. However, some of them quickly become intractable. The reason for this is that a naive approach to calculating the metrics would be to search across the entire configuration space of the leg, despite really only requiring calculations for realistically plausible footholds. Searching the entire configuration space could be executed by incrementing each joint from its lower limit to upper limit at a set resolution to capture all combinations of joint configurations at the given step resolution. At each of these sampled robot configurations the pose of any point on the leg (such as the foot) can be obtained with forward kinematics. Unfortunately, sampling this way at a meaningful resolution makes calculating a design metric intractable. To illustrate, consider a metric for a 16-DoF configuration space sampled at a resolution of 10° (i.e., 18 samples per joint variable in the case of a rotatory joint moving from −90° to 90°). Calculating this metric would require 2.884 × 10^20^ calculations. If a computer could compute each of these calculations in 10^–12^ s, it would still take over 9 years to solve. Additionally, a resolution of 10° is generally too coarse to be useful, especially for the calculation of the metrics presented in this paper. Note that this 9 year computation time is only for a single design. A fully-developed optimization will repeat this calculation thousands of times. In this section we introduce a method to make this approach possible while maintaining a sufficiently fine resolution.

#### 3.3.1 Description

To emphasize an important point we must restate that this generalization enables searching over only the possible footholds of a legged robot instead of the entire configuration space, which is far more tractable.

**FIGURE 3:**
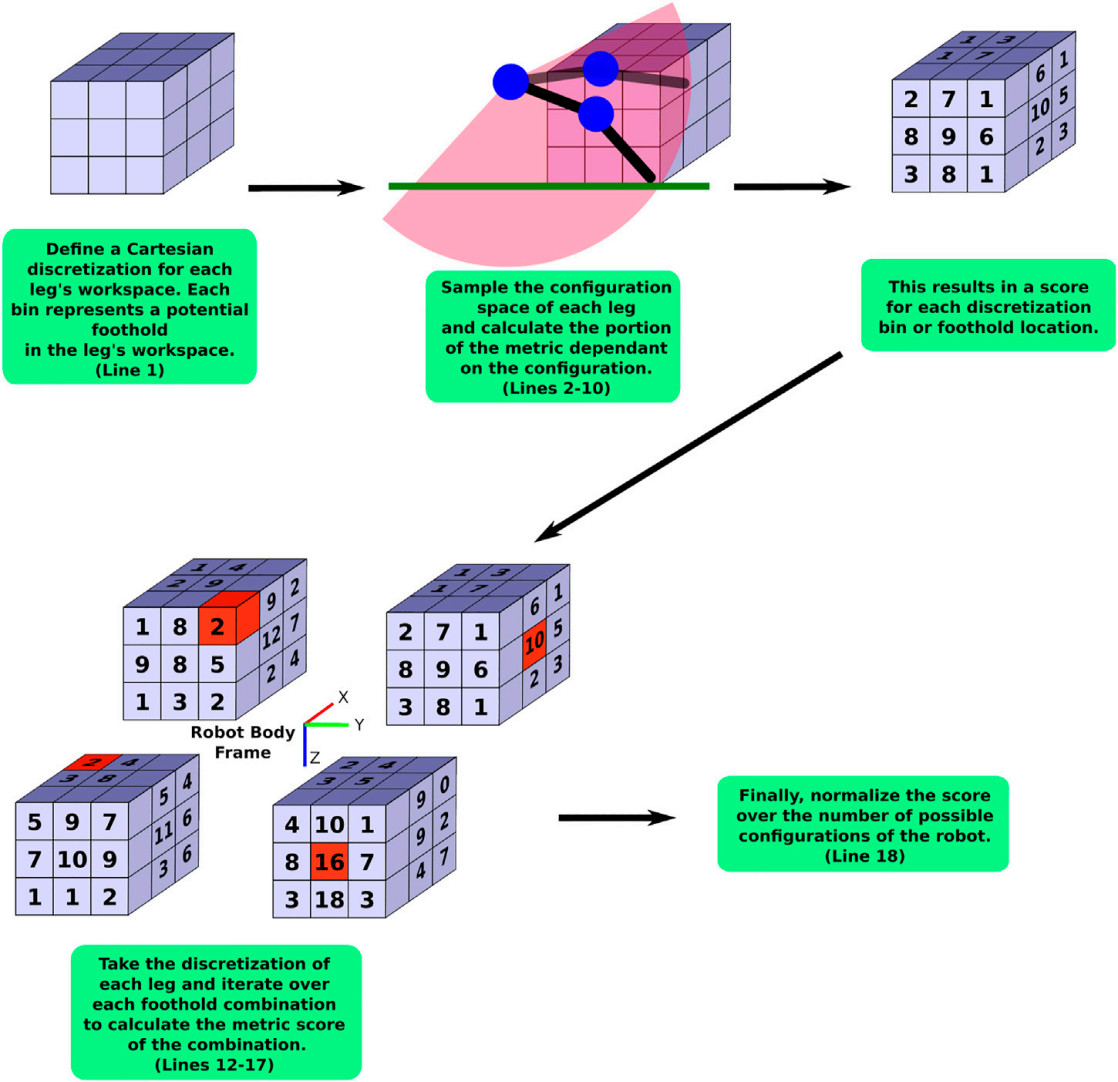
A flow chart visualizing the general approximation method as outlined in detail in Algorithm 1. The red cubes indicate an example of a foothold combination.

Algorithm 1 shows this general approximation method and Figure 3 shows a flow chart to help visualize the main parts of this algorithm. First, a discretization of the Cartesian workspace of each leg is defined (Line 1). This can be either a full 6D pose discretization or a 3D/2D position-only discretization. Next, each of the legs’ configuration spaces are sampled in Lines 2–3. Whenever the leg’s foot is located within the desired walking region *W* (as described in Section 3.1), we calculate the portion of the metric that is specific to the leg configuration, *s*_*C*_ (Lines 4–5). For the *average static stability* metric detailed in Section 3.4, *s*_*C*_ is the horizontal discretized bin location of the foot for the average static and longitudinal stability margins. For the *average desired velocity* metric in Section 3.5, *s*_*C*_ is calculated on Lines 6–10 of Algorithm 3.

On Line 6, we combine *s*_*C*_ of the current leg configuration with the other *s*_*C*_’s that were found from other configurations that reached the same discretization bin. This results in a score for the specific discretization bin, *s*_bin,*j*_. As an example, the *average desired velocity* metric (in Section 3.5) always takes the lowest score found in the bin since this is the limiting factor on the desired velocity. On Line 7, we record the number of times, *n*_bin,*j*_, the leg reaches the *j*th discretization bin.

Algorithm 1 General Legged Robot Metric Over Configuration Space Approximation

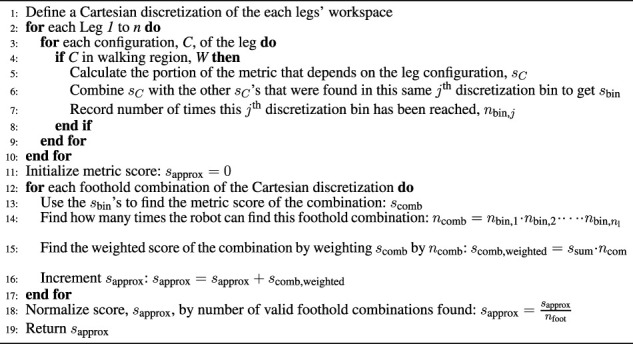

Once each leg’s configuration space is sampled to calculate *s*_bin_ for each discretization bin (i.e. *s*_bin,*j*_, *∀j*), we iterate over each of the possible combinations of discretization bins from each leg (Line 12). For each combination of footholds, we calculate the part of the metric that depends on the combinations of all the footholds, *s*_comb_ (Line 13). We then sum all the *s*_comb_ together (Line 16) and then normalize this sum by the number of possible combinations of legs, *n*_foot_ in *W* (the *dexterity in walking region* metric as calculated in Section 3.1.2) in Line 18 to arrive at the approximation of the metric.

The number of calculations for the approximation can be simplified further by projecting the spatial discretization onto a 2D discretization (Lines 11 and 12) as we do in Sections 3.4 and 3.5. This is done by combining the scores of 6D or 3D bin scores into a single 2D discretization bin score in a meaningful way. For this paper, we average all the scores in bins that are being lumped into a single 2D bin. This simplification is valid as long as the lumping of bins into 2D bins is still meaningful for the metric. Both of the metrics we describe next are only looking for averages, so this simplification is reasonable. It is also reasonable for any metric that is looking for an average over the entire workspace.

The tractability of this approximation is dependent on the number of distinct bins in the spatial discretization used for iterating over the foothold combinations (Line 12). If there are too many bins, this approximation method will exhibit the same problems as sampling the entire legged-robot’s workspace. However, it can be far more tractable for meaningful discretizations than attempting to search the full configuration space of a high-DoF legged robot. For example, in our application (described in more detail in Section 4), we calculate approximate metrics of the 16-DoF quadruped by sampling the configuration space at a resolution of 4° (i.e., 45 increments per joint variable) using 2D Cartesian square bins that are 10 cm wide in the approximations. We experimentally observed that this calculation takes at most a few hours to solve, while sometimes it was solved in as little as 20 s. Contrast this to the example presented at the beginning of this section where sampling the full configuration space of the 16-DoF robot once using only 10° increments would take over 9 years to calculate the objective function once.

#### 3.3.2 Validation

We validate the configuration space approximation presented in this section with gradient-based optimizations for both the *average static stability criteria* and *average desired velocity* metrics and their respective approximations on a simulated four-DoF quadruped robot. Additional details about this validation are included in Sherrod (2019). However, in this paper we simply outline the validation method and result in order to give confidence that this approximation is both useful (i.e., computationally) and accurate.

**FIGURE 4:**
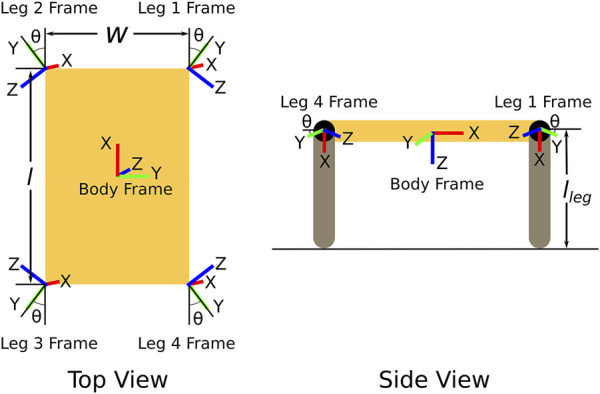
Top and side view of the simple four-DoF quadruped design.

Figure 4 is a diagram of the four-DoF quadruped we used for validation experiments. It consists of a base of length *l* and width *w*. The four legs of length *l*_leg_ are attached to the corners of the base via rotary joints which rotate about the *z*-axis of each leg frame. The mount angle, *θ*, indicates the angle at which these joints are attached relative to the base. The robot is designed to be symmetric about the body frame’s *x* and *y* axes. We acknowledge that this four-DoF quadruped may not be efficient–or even able to walk. But its simplicity allows our design metrics to be calculated directly and then compared to their approximations.

The design space for the stability margin optimizations consisted of *w*, *l*, and *θ* while the design space for the *average desired velocity* metric was only composed of *θ*. This is because changes in *w* and *l* do not affect the *average desired velocity* metric.

Results for these optimizations are shown in Table 3. All the metrics obtained by using the approximation of the configuration space result in the same optimum as the metric which used the entire configuration space. The highest error is for the *average desired velocity* approximation and is only −1.01 × 10^–2^ degrees.

The important outcome here is that the proposed approximation matches the gradient trends [see Sherrod (2019)], and solutions from a less tractable but more accurate gradient-based method. This gives confidence that this method is a valid approach to optimizing a 16 degree-of-freedom continuum soft robot as detailed in Section 4.

### 3.4 Average Static Stability Criteria

#### 3.4.1 Metric Definition

There are many static-gait controllers and/or planners for robots with four or more legs that use a measure of stability in a given configuration to plan and execute gaits. These measures are referred to as stability margins. Several of these stability margins are surveyed in De Santos et al. (2007). Configurations with larger stability margin values are deemed more stable and therefore, more desirable in planning and control. Designing a robot to maximize these stability margins over the entire configuration space eases the implementation of the aforementioned controller. We therefore use the *average static stability criteria* which is the average stability margin over all of the robot’s valid configurations as an optimization metric. Here, valid configurations are defined as configurations where three or more legs can be found within the walking region *W* (i.e., one of the foothold combinations counted in *n*_foot_ as described Section 3.1).

We choose to use two of these stability margins in our experiments: the static stability margin originally from McGhee and Frank (1968) and the longitudinal stability margin originally from McGhee and Iswandhi (1979). As will be shown in Section 3.4.2, these specific margins are chosen because we can simplify their calculation to make the optimization tractable. Also, they follow the same trends as the other stability measures as seen in the experiments in De Santos et al. (2007). Therefore, maximizing one of these tends to maximize the other average stability measures as well.

**FIGURE 5:**
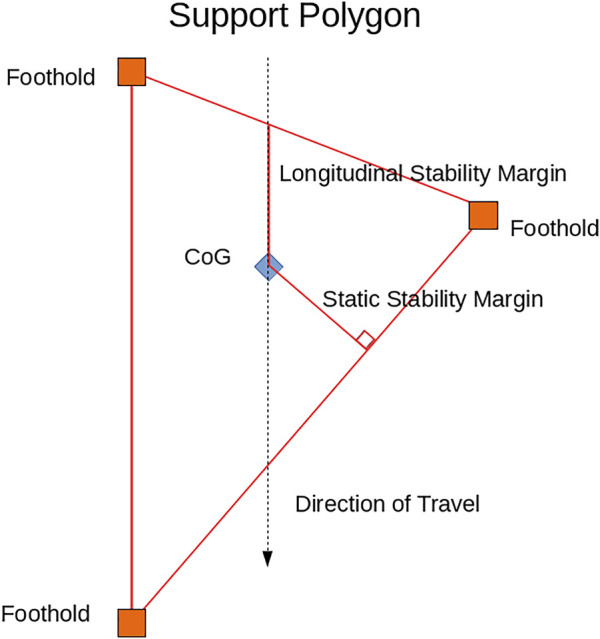
Visualization of the support polygon and the static stability margin and longitudinal stability margin.

The basis of the static stability margin and longitudinal stability margin are as follows. For static gaits, the horizontal projection of the center of gravity of an ideal legged robot (one with massless legs) must remain within the support polygon to remain stable. The support polygon is defined as the convex hull about the contact points projected onto a horizontal plane. Figure 5 illustrates this for a robot with three legs making contact with the ground. The static stability margin is the shortest distance from the center of gravity projection to the edge of the support polygon. The longitudinal static stability margin is the shortest distance from the center of gravity projection to the edge of the support polygon along a given direction which usually corresponds with the direction of intended travel. Both of these margins are diagrammed in Figure 5. While these measures only guarantee stability for an ideal robot whose legs are massless, they do provide insight into the stability of real-world robots whose legs cannot be modeled as massless.

#### 3.4.2 Metric Approximation

**FIGURE 6:**
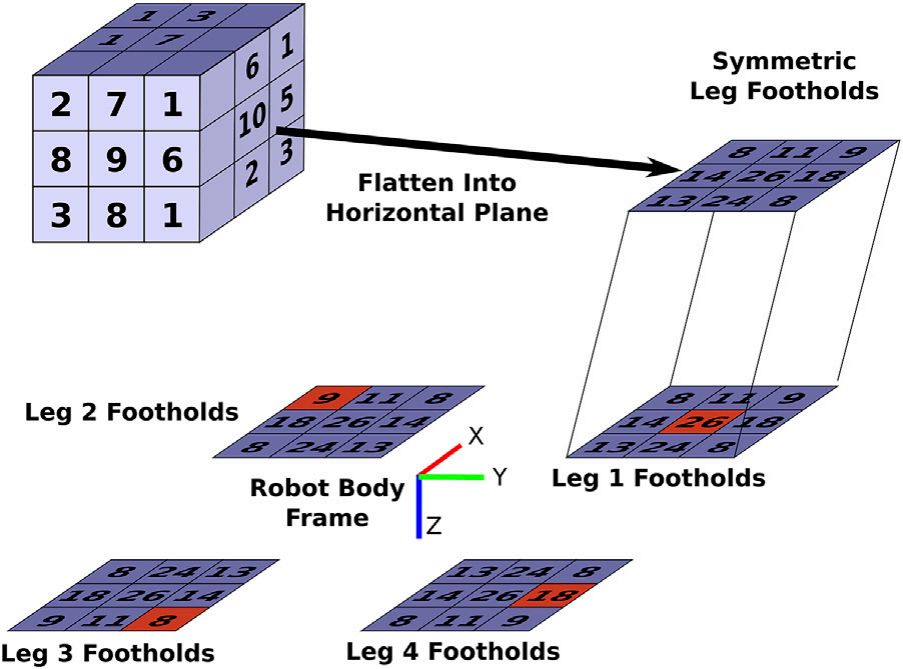
Illustration of the calculation of the approximation of *average static stability criteria metric*. The highlighted red squares indicate an example sampling of the possible footholds of the quadruped from its four legs.

We approximate the *average static stability criteria* as follows for the quadruped. First, recall that the calculation of the *dexterity in walking region* metric (Section 3.1) results in the 6D discretization of the foot’s pose whenever the foot is found within *W*. We record the number of distinct orientation bins reached at each Cartesian bin. The top-left corner of Figure 6 is a simple illustration of this. For example, in the bottom-left Cartesian bin, three distinct orientation bins are reached. We then flatten this 3D Cartesian discretization into a 2D Cartesian discretization that is a horizontal plane parallel to the *xy* plane of Robot Body Frame as shown in Figure 6. This is done by adding the orientation counts of each discretization bin that is located above a given bin in the *xy* plane to find the total number of orientations for the single bin representing the entire column in the 2D discretization. We then mirror this 2D Cartesian bin about the Robot Body Frame for each leg of the quadruped the symmetric leg represents as illustrated in Figure 6. Note how symmetry is preserved about the Robot Body Frame with this operation. This transformation of the 2D Cartesian bins for each leg is necessary since we need the locations of the feet for each of the four legs to calculate the stability margin. Enforcing symmetric designs simplifies the problem as we only need to find the possible footholds of one leg and then transform these footholds to the locations of the other legs instead of having to find the possible footholds of all four legs separately.

Algorithm 2 Average Static Stability Criteria Approximation Calculation

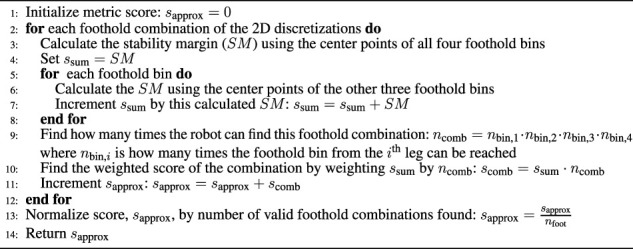

We then calculate the metric approximation by iterating through each possible foothold combination of the four feet in the 2D horizontal Cartesian bins as described in Algorithm 2. An example of one of these combinations is highlighted in red in Figure 6. We calculate the stability margins for each of these combinations using the center of the 2D Cartesian bins for the footholds’ position. As shown in Algorithm 2, we look at the stability margin for when all four feet are in contact with ground (Line 3) as well as the four cases for when only three of the feet are in contact with the ground at these horizontal positions (Line 6). This is a total of five different stability margin calculations for each combination of 2D bins (which corresponds to the combination formula sum of Eq. 1). The weighted stability score for each foothold combination, *s*_comb_, is found by multiplying the sum of these five stability margins, *s*_sum_, by the number of times a particular combination of the 2D bins can be found in the overall configuration space, *n*_comb_ (Line 10). This weighting is necessary since we desire the average stability margin, and therefore, we need to weight the scores of the foothold combinations according to the frequency the robot can find them. This frequency, *n*_comb_, is found on Line 9. Here, *n*_bin,*i*_ is the number of orientations for the *i*th bin which corresponds to the foothold bin in the combination from the *i*th leg. As an example, for the foothold combination highlighted in red in Figure 6, *n*_comb_ is *n*_comb_ = 9 ⋅ 26 ⋅ 8 ⋅ 18 = 33, 696. In Line 11, all of the *s*_comb_ are added together and normalized by the total number of valid foothold combinations, *n*_foot_, (the *dexterity in walking region* metric described in Section 3.1.2) to obtain the approximated *average static stability criteria*, *s*_approx_.

While this approximation is shown for a quadruped robot with symmetric legs. It can easily be extended to a non-symmetric robot where each leg’s workspace is binned individually and flattened into a 2D discretization. It can also be expanded to robots with *n* number of legs where for each combination of 2D bins, the stability margin is calculated for each possible combination where there are at least three footholds.

The tractability of this approximation is clearly dependent on the number of Cartesian discretization bins. During computation the time spent iterating through combinations of 2D horizontal Cartesian bins is much larger than that of searching the configuration space of a single leg. Therefore, extending this metric to non-symmetric legs is likely more tractable than extending this to more legs.

### 3.5 Average Desired Velocity

#### 3.5.1 Metric Definition

While the *average static stability criteria* metric indicates a robot’s ability to find stable configurations, it does not ensure that the robot will be able to apply the necessary forces on the ground to propel itself in a desired direction. The *average desired velocity* metric helps indicate this ability.

To walk in a desired direction, a leg needs to be able to apply a sufficient force opposite this direction to propel the body forward. The leg’s ability to apply this force is composed of two parts:

1. Movement in the necessary direction to apply the force.
2. Execution of the joint efforts required to apply the necessary force in that direction.

The *average desired velocity* metric analyzes a robot’s average ability to accomplish this over all of its valid configurations. Here, valid configurations are defined as configurations where three or more legs can be found within the walking region, *W* (one of the foothold combinations found to calculate *n*_foot_ as described in Section 3.1).

An indication of a leg’s ability to move in a given direction can be found via its manipulator Jacobian (which is a function of leg’s configuration). The particular Jacobian of interest is the one that relates the joint velocities of the leg to the Cartesian velocities of the foot expressed in the body frame of the robot.

The top three rows of this Jacobian indicate the effect of the joint variables on the foot’s movement in the *x*, *y*, and *z* directions of the body frame. The sum of the absolute values of each element of these rows indicates the maximum velocity at which the foot could move in each of these three body frame directions. Thus, maximizing these values for a particular direction over all of a leg’s configurations would increase its ability to move in this desired direction. However, movement alone does not guarantee that the leg is capable of applying the necessary force. Given a configuration, a leg must be capable of applying the necessary force to propel the robot forward in this direction.

#### 3.5.2 Metric Approximation

Algorithm 3 Average Desired Velocity Metric Approximation Calculation Part 1

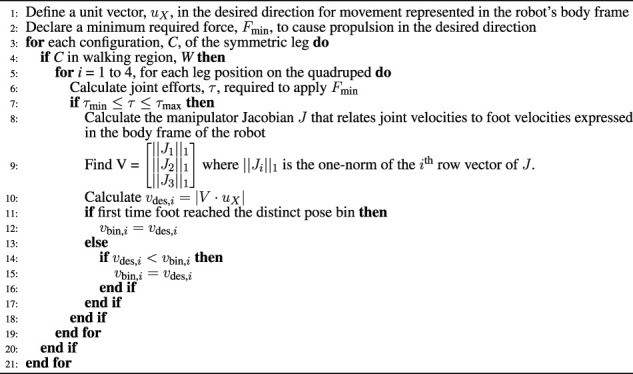

The approximation for the *average desired velocity* for the symmetric robot is calculated in three parts:

1. Sampling the leg’s configuration space to assign scores to the pose bins of the walking region, *W*, which is shown in Algorithm 3.
2. Combining the scores of pose bins in a 3D column of the discretization to create a 2D discretization and then transforming these 2D discretizations into the Robot Body Frame (similar to what is shown in Figure 6 for the stability metric approximation).
3. Iterating through the possible foothold combinations to calculate the approximation of the *desired velocity* metric as outlined in Algorithm 4.

In Algorithm 3, we show how to sample the leg’s configuration space (Line 3). Each time the foot is found in the walking region (Line 4), the portion of the metric that is dependent on the configuration of the leg is calculated for each leg (one through four) of the quadruped (Lines 5–19). This allows us to get scores for each pose bin in the discretization of *W* for all four of the legs (*v*_bin,__1_, *v*_bin,__2_, *v*_bin,__3_, and *v*_bin,__4_) with one sampling of the configuration space. As is seen in Lines 11–17, the lowest score found in a distinct pose bin is the one that is recorded. This is because the lowest score correlates to the limiting configuration on possible velocities in the bin. Therefore, using the lowest score guarantees that all configurations reached in the bin can at least achieve this desired velocity (with the assumption the leg can provide the torque necessary for acceleration as mentioned in Section 3.5.1).

The calculation of the joint efforts, *τ*, required to apply *F*_min_ (Line 6) is approximated in a similar manner to the calculation of the joint efforts required to support the robot’s weight for the *average payload in walking regions* metric in Section 3.2.1. *F*_min_ is simply added to the FBD which results in Eq. (5) becoming

$$\tau =\overset{n}{\underset{i=1}{\sum }}\left(J_{\text{link,}i}^{T}m_{\text{link,}i}g+J_{\text{joint,}i}^{T}m_{\text{joint,}i}g\right)+J_{\text{foot}}^{T}\left(N+W_{\text{min}}\right)$$ (10)

where *W*_min_ is *F*_min_ represented as a 6 × 1 wrench with the torques being set to zero. Note, *F*_min_ is simply a possible value of the friction force, *f* from Eq. 5.

Algorithm 4 Average Desired Velocity Metric Approximation Calculation Part 2

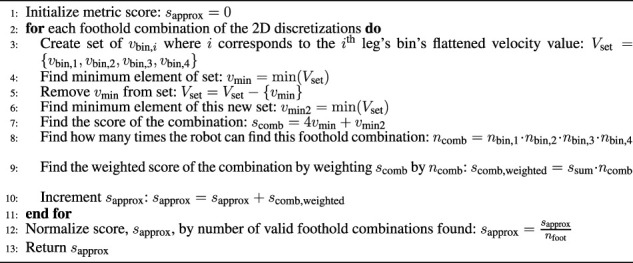

After the *v*_bin,*i*_ is calculated for every orientation bin reached by each leg, the 6D pose discretization is flattened to a 2D discretization similar to what is shown in Figure 6. However instead of adding the scores, as we did for the approximation of the *average stability criteria* metric, all of the *v*_bin,*i*_ values of each distinct 6D pose bin are averaged to find the *v*_bin,*i*_ for the 2D rectangular bin. Since the goal is the *average desired velocity* metric, taking the average finds the average performance in the 2D bin. We translate this flattened discretization into the Robot Frame for each of the four legs as is done for the approximation of the *static stability criteria* metric in Section 3.4.2 (visualized in Figure 6).

We finally iterate over each combination of four footholds (one foothold from each leg) in the 2D horizontal Cartesian discretizations, similar to what is described in Section 3.4.2 and Figure 6, to approximate the portion of the metric that depends on the results of all the legs together. This is outlined in Algorithm 4. For each combination, we create the set of *v*_bin,*i*_’s which are the *desired velocity* scores for each 2D bin of the combination. We find the two smallest values of the set in Lines 4-6 and use these to calculate the *desired velocity* score of the foothold combination (*s*_comb_) in Line 7. This results in *v*_min_ being used for four of these combinations when it is the limiting factor and *v*_min2_ being used for the combination of three footholds when the foot causing *v*_min_ is lifted.

We weight the combination score, *s*_comb_, by the number of times the foothold combination can be found in the overall configuration space, *n*_comb_, in Line 9 since we want to find the overall average. This weighting, *n*_comb_, is calculated in the same manner as it is for the approximation of the *average static stability criteria* metric in Line 8. We sum all of the *s*_comb_ for each foothold combination together (Line 10) and then normalize them by the number of valid foothold combinations, *n*_foot_, (Line 12) to obtain the approximated *average desired velocity* metric, *s*_approx_.

While this approximation is shown for four symmetric legs, like the approximation for the *average static stability criteria* metric, this can easily be extended for a non-symmetric *n*-legged robot. However, it has the same limitations in tractability as the approximation for the *average static stability criteria* mentioned in Section 3.4.2.

## 4 Optimization of 16-DoF Continuum-Joint Quadruped

In this section we demonstrate how the application of the proposed metrics and their approximations (from Section 3 allow us to optimize the design of a 16-DoF continuum joint quadruped. We also present and explore trade-offs between the metrics to show how these tools may be used in the design process and which metrics or objectives are competing.

### 4.1 Robot Description

**FIGURE 7:**
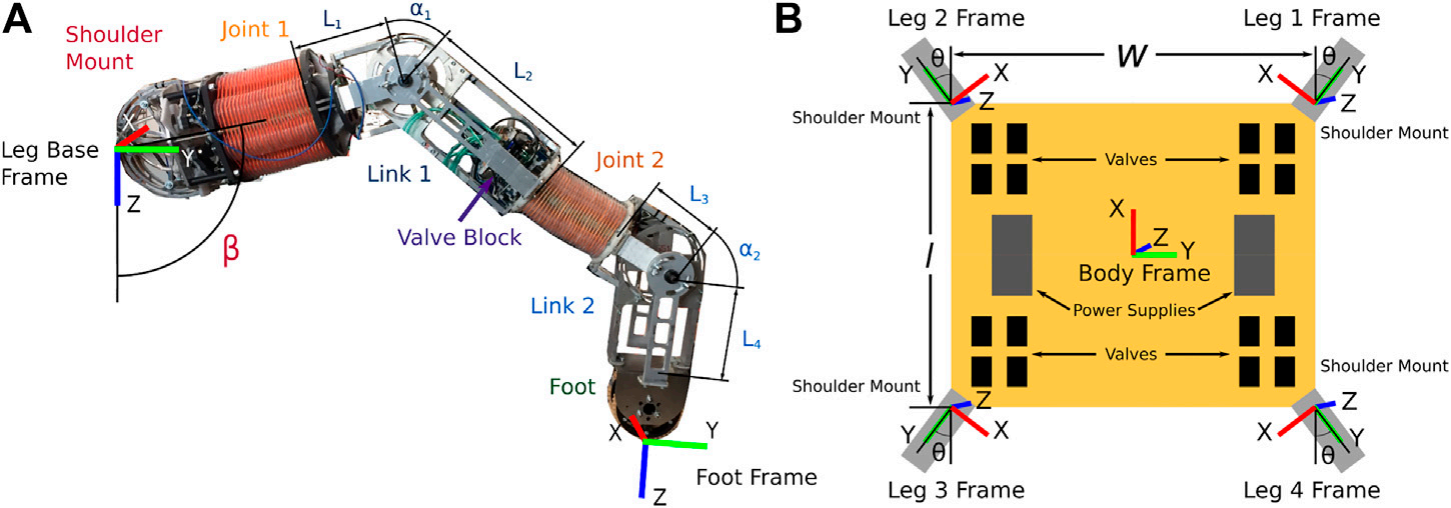
**(A)**: An illustration of the leg of the robot. Joint 1 is a two-DOF, eight-bellows, pneumatically-actuated, continuum joint. Joint 2 is a two-DoF, four-bellows, pneumatically-actuated, continuum joint. The valve block actuates Joint 2. A foot is attached to the end of Link 2. **(B)**: A top view of the model of the body of the quadruped. Valves needed to actuate Joint 1 are included in this model.

The design of the 16-DoF quadruped consists of a wooden base and four individual four-DoF, pneumatically-actuated, continuum-joint legs attached to the base. The robot is designed to be symmetric to reduce the number of design variables and to simplify the calculation of the metrics presented in Section 3. Illustrations of the leg design and base are shown in Figure 7. In total, we have ten design variables for the 16-DoF quadruped: $x=\left[w,l,\theta ,\beta ,L_{1},L_{2},\alpha_{1},L_{3},L_{4},\alpha_{2}\right]$ . Their descriptions are listed here:

• Base width *w*.
• Base length *l*.
• Mounting angle of the legs *θ*.
• A shoulder mount attaching the leg to the base positioned at an elevation angle *β*.
• A link (called Link 1) defined by lengths, *L*_1_ and *L*_2_, and bend angle, *α*_1_.
• A link (called Link 2) defined by lengths, *L*_3_ and *L*_4_ and bend angle, *α*_2_.

Each leg of the quadruped design consists of two separate two-DoF, pneumatically-actuated, continuum joints as seen in Figure 7. These joints operate by using two sets of antagonistic bellows that can be filled to pressures ranging from zero to 600 kPa (gauge). There are two versions of these joints: one with four bellows and one with eight. They are identical in function with the exception that the eight-bellows version is larger and produces more torque. Each joint is modeled as having two degrees of freedom (denoted *u* and *v*) and we refer readers to Hyatt et al. (2020) for more in-depth discussion of the kinematic and dynamic properties of these soft actuators.

### 4.2 Optimization Problem

Our multi-objective optimization problem is formally stated as

$$\begin{array}{c} \underset{x}{\text{max}}\;F\left(x\right)=\left[f_{1}\left(x\right),f_{2}\left(x\right),f_{3}\left(x\right),f_{4}\left(x\right)\right]\\ \text{subject to}\;L_{1}+L_{2}+L_{3}+L_{4}+2h+L_{\text{foot}}-L_{\text{max}}\leq 0\\ {\underline{x}}_{i}\leq x_{i}\leq {\bar{x}}_{i}\;i\in \left[1,10\right] \end{array}$$ (11)

where $x\in R^{10}$ is the vector of design variables described in Section 4.1. *F*(*x*) is a vector containing each of the four design metric approximations outlined in Section 3. *h* is the length of the joints (0.205 m) and *L*_max_ = 1.829 m (6 ft). The first inequality constraint prevents overly long legs. In the second inequality, ${\underline{x}}_{i}$ and ${\bar{x}}_{i}$ are the *i*th design variable’s lower and upper bounds respectively. Numerical values for the bounds are given in Table 1. We choose to solve this optimization problem with a genetic algorithm adapted from Bodily (2017) to the quadruped design. *F*(***x***) is the vector-valued objective function where *f*_*i*_(***x***) is each of the four design metrics discussed in Section 3. The objective function is evaluated for each point in the population and converted to an individual fitness score using the maximin fitness function presented in Balling and Wilson (2001) in order to find an approximate Pareto Frontier. Algorithm 5 shows our implementation of the evolutionary algorithm.

**TABLE 1:** The bound constraints and perturbation bounds for each design variable of the 16-DoF continuum-joint quadruped.

Design Variable	Lower Bound	Upper Bound
*w*	0.4 m	1.3 m
*l*	0.4 m	1.3 m
*θ*	−45°	135°
*β*	−180°	0°
*L*_1_	0.185 m	0.5 m
*L*_2_	0.263 m	0.5 m
*L*_3_	0.143 m	0.5 m
*L*_4_	0.150 m	0.5 m
*α*_1_	−90°	90°
*α*_2_	−90°	90°

Algorithm 5 Evolutionary Algorithm Implementation

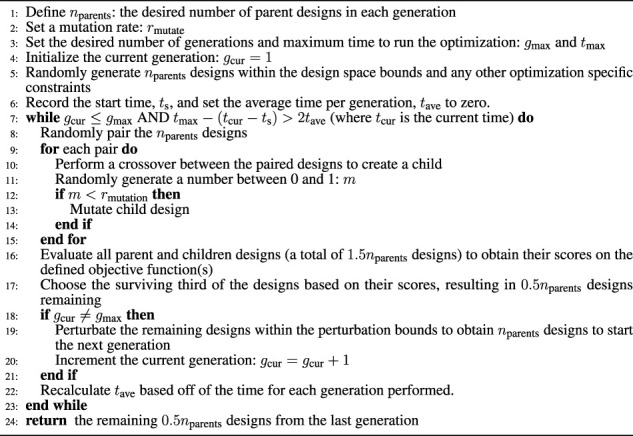

Table 2 lists the fixed parameters that were used to calculate *F*(***x***). This includes the *XYZ Discretization Bin Size* and *SO3 Discretization Bin Size* which are the resolutions of the discretization of the workspace. We sample the leg’s configuration space at every combination of *u* and *v* for each joint between $-\frac{\pi }{2}$ and $\frac{\pi }{2}$ at the resolution *Joint Angle Res*. However, because of physical joint limits, the magnitude of the vector formed by *u* and *v* cannot exceed $\frac{\pi }{2}$ . If this constraint is violated in a given configuration the design metrics are not calculated. This ensures only realistic leg configurations are included in the population. *Min Walking Clearance*, *Max Walking Clearance*, and *γ*_max_ are the parameters necessary to define the walking region, *W*, which is needed to calculate all of the design metrics. As mentioned in Section 3.2.1 *F*_payload,_ _max_ is necessary to calculate the *average payload in walking regions* metric and is chosen based on the robot structure such that it is lower than the maximum allowable buckling, axial, or shear force.

The vector ${\vec{u}}_{x}$ in Table 2 is the unit vector indicating the desired direction in the quadruped’s Body Frame (see Figure 7) used to calculate the *average desired velocity metric*. The vector ${\vec{F}}_{\text{min}}$ is necessary in the calculation of the *average desired velocity metric* (see Section 3.5). *μ*_*s*_ is the static coefficient of friction between the foot and carpet (equal to 1.8 between the foot and the carpet in our lab) and *N* is the magnitude of the normal force for a single leg described in Section 3.2. In that section, *N* is defined as half the weight of the robot, and therefore, it is a function of the quadruped design being evaluated. The direction, $-{\vec{u}}_{x}$ , ensures this force provides propulsion in the desired direction. The magnitude *μ*_*s*_ *N* represents the maximum possible force the robot can apply without slipping and results in the max possible acceleration of the quadruped. Since ${\vec{F}}_{\text{min}}$ is the minimum force that a leg configuration must be able to apply to include its score in the *desired velocity* metric (refer to Section 3.5.1), it does not make sense to require this minimum force to provide the maximum possible acceleration for the leg. Thus, we chose a value for ${\vec{F}}_{\text{min}}$ that was an order of magnitude lower than this force (i.e. 0.1). In other words, it is acceptable to include designs that are not able to provide maximum acceleration in the *desired velocity* metric.

**TABLE 2:** A list of fixed parameters for the optimization of the 16-DoF continuum-joint quadruped.

Parameters	Value
Min Walking Clearance	0.25 m
Max Walking Clearance	0.8 m
*γ*_max_	45°
Joint Angle Res	4°
XYZ Discretization Bin Size	0.20 m
SO3 Discretization Bin Size	4.93°
*F*_payload,_ _max_	981 *N*
${\vec{u}}_{x}$	$\left[\begin{array}{c} 1and0and0 \end{array}\right]$
*μ*_*s*_	1.8
${\vec{F}}_{min}$	$-0.1\mu_{s}N\vec{u_{x}}$

**TABLE 3:** Optimization results for four-DoF quadruped. Values marked by * indicate values that were kept constant for the particular optimization.

Metric	*w*_start_	*l*_start_	*θ*_start_	*w*_opt_	*l*_opt_	*θ*_opt_
Static Stability Margin	1.0	1.0	30.0	1.25	1.25	−45.0
Approx. Static Stability Margin	1.0	1.0	30.0	1.25	1.25	−45.0
Error	-	-	-	7.0*e* − 11	1.0*e* − 10	−1.0*e* − 11
Longitudinal Stability Margin	1.0	1.0	30.0	0.125	1.25	−45.0
Approx. Longitudinal Stability Margin	1.0	1.0	30.0	0.125	1.25	−45.0
Error	-	-	-	2.0*e* − 12	0.0	2.0*e* − 12
Average Desired Velocity	0.1*	0.2*	30.0	0.1*	0.2*	−8.88*e* − 3
Approx. Average Desired Velocity	0.1*	0.2*	30.0	0.1*	0.2*	1.25*e* − 3
Error	-	-	-	-	-	−1.01*e* − 2

### 4.3 Results

We initialize the genetic algorithm with 200 random quadruped designs. Per the evolutionary algorithm outlined in Bodily (2017), this results in 100 designs surviving from one generation to the next based on their maximin fitness function values. We choose to preserve a large number of designs from one generation to the next to clearly delineate the 4-dimensional Pareto front. The objective function for each design in a generation is parallelized on a supercomputer with 28 cores on a 14-core Intel Broadwell (2.4 GHz) processor. The optimization took 1 day, 15 h, and 40 min to evolve 60 generations. Between all the cores, there was over 46 days of computation time. In total, we ran the optimization ten different times varying the population size. We did not observe meaningful differences between the resulting Pareto fronts, so the results presented in this section are from a single representative trial. The data for all optimization runs is publicly available at the link provided in the Data Availability Section.

**FIGURE 8:**
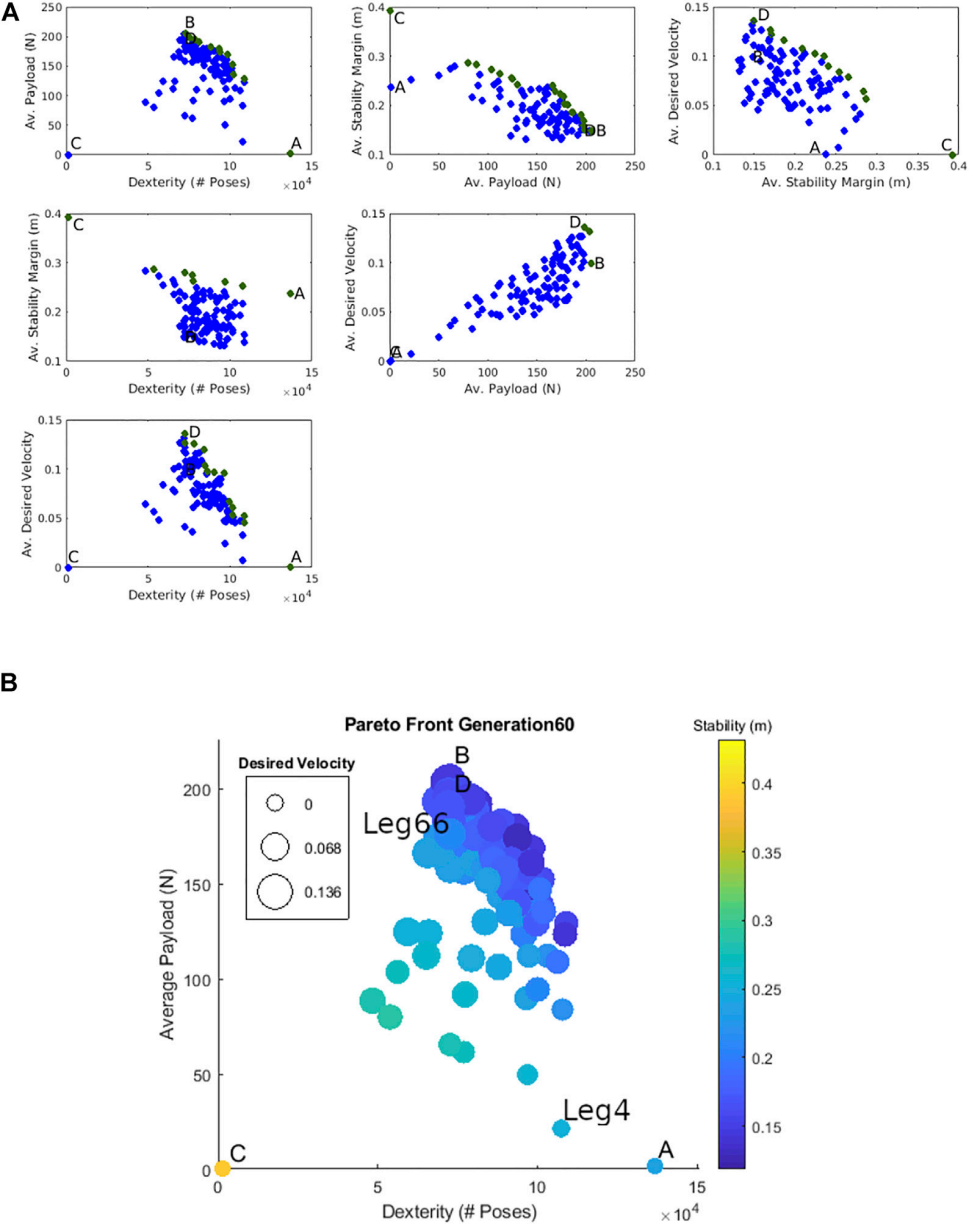
**(A)** The Pareto front of the optimization after 60 generations represented in a scatter plot matrix. The robots with the best dexterity in walking region, average payload, average stability criteria, and averaged desired velocity are labeled as A, B, C, and D respectively. **(B)** The Pareto front of the optimization after 60 generations. The robots with the best dexterity in walking region, average payload, average stability criteria, and averaged desired velocity are labeled as A, B, C, and D respectively.

The optimization was terminated on the 60th generation when the Pareto front converged. We define convergence in this context to mean that the top-scoring designs for each metric along the Pareto front improve less than 1% for ten consecutive generations. For comparison, we observed these four individuals improve between 15 and 363% in each metric from the first generation. Figure 3.14 of Sherrod (2019) also shows the progression of this Pareto front every ten generations. Figure 8B shows the Pareto front of the last generation. The four objectives are shown in bubble plots with the *dexterity in walking regions* and *average payload in walking region* metrics represented on the *x* and *y* axes, respectively. The *average stability margin* metric is represented by the color of the bubbles and the *average desired velocity* metric is represented by the bubble size. Recall that we are maximizing the objective function (Eq. 11) so larger metric values are better.

For comparison, the designs with the best *dexterity in walking region*, *average payload*, *average stability criteria*, and *averaged desired velocity* are labeled as A, B, C, and D respectively.

Generally, designs that do well in stability perform poorly in the other three metrics. The prime example of this is that the quadruped with the best stability score (C) has a score of zero in the *average payload* and *average desired velocity* metrics and a low score for the *dexterity in walking regions* metric. Also note that designs that perform well in the *average payload* also perform well in *desired velocity*–as is seen by the proximity of B and D. This is most likely because we require a design to be able to produce enough force to carry its own weight and accelerate forwards in the calculation of the *average desired velocity* metric. Thus robots that are able to supply the necessary force in the desired direction are more likely to be able to support higher payloads as well.

Figure 8A helps further illustrate the relationships between the different objectives. Here, the final population of designs for the optimization in four-dimensional objective space are plotted in the six possible pairs between the four objectives (known as a scatter plot matrix). This shows how any two of the four metrics are related to each other. Those designs that are non-dominated with respect to the two metrics in a given plot (i.e., Pareto optimal) are marked in green. A, B, C, and D are also marked in each plot. As is indicated by the near-vertical slope of its Pareto front, the only non-competing objective is *average payload* and *average desired velocity* metrics. All other Pareto fronts have negative slopes, indicating competing objectives.

**FIGURE 9:**
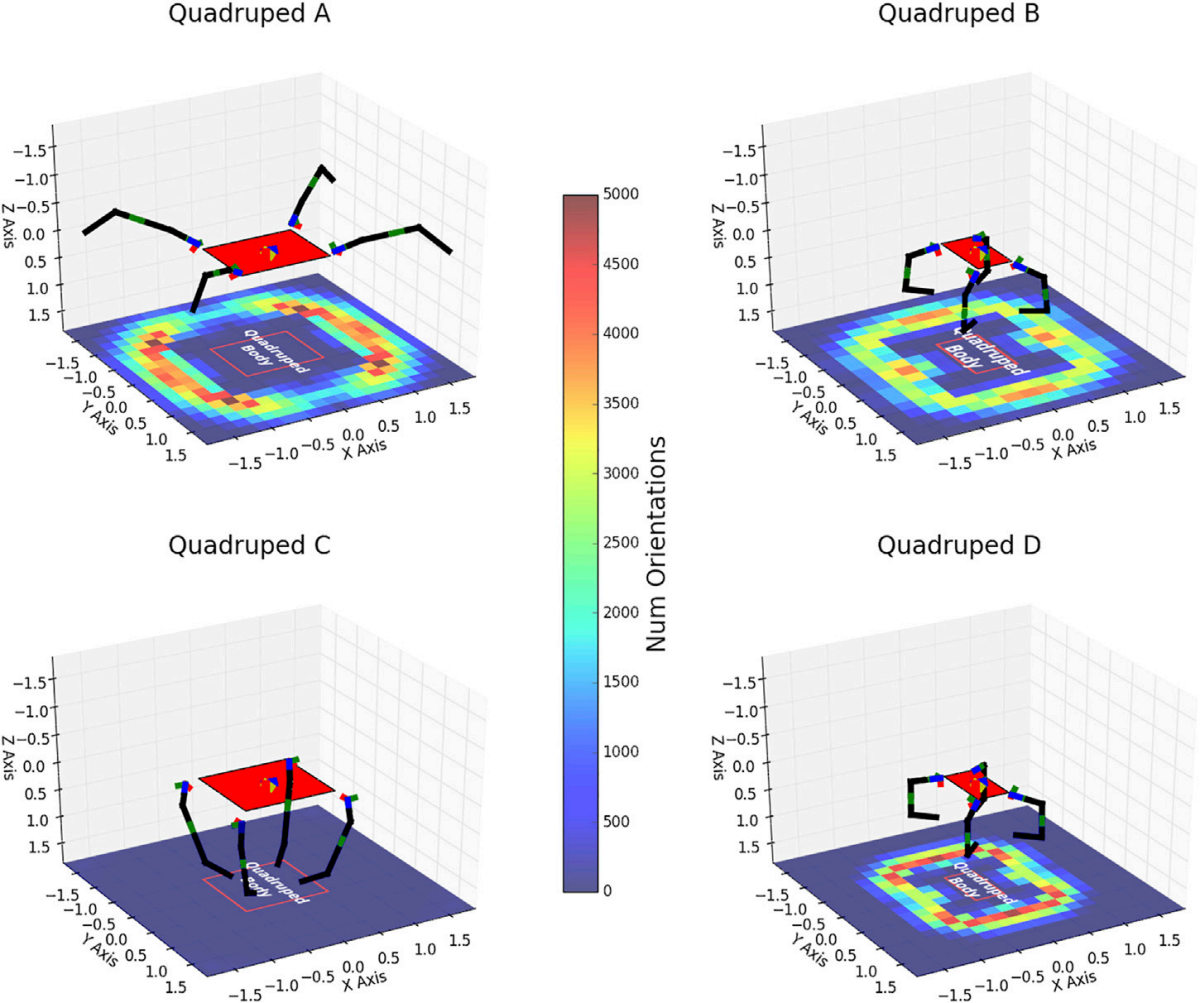
Visualization of designs (A–D). Axis units are in meters. Rigid links are shown in black while the pneumatic joints are shown in green. The red outline on the heat map is a projection of the quadruped body (solid red) onto the *x* − *y* plane for visualization clarity.

Table 4 lists the design variables of A, B, C, and D marked in Figure 8A,B. Figure 9 shows these quadruped designs in their zero configurations (i.e., *u* and *v* set to zero) over heat maps indicating how many foot orientations exist in a given horizontal 2D bin location in the body frame. This helps to illustrate each quadruped’s dexterity and stability performance. These plots are similar to what is described and illustrated in Figure 6. The red rectangle in these figures indicates the outline of the quadruped’s body.

**TABLE 4:** Kinematic values for the labeled designs in Figures 9, 10. Lengths are in meters, and angles are in degrees. Leg66 and Leg4 are used for hardware experiments in Section 5.

Design	*w*	*l*	*θ*	*β*	*L*_1_	*L*_2_	*L*_3_	*L*_4_	*α*_1_	*α*_2_
A	0.953	1.234	37.8	−124.6	0.185	0.382	0.145	0.369	12.4	68.4
B	0.905	0.471	37.8	−59.9	0.185	0.263	0.143	0.150	55.1	90.0
C	1.158	1.239	45.6	−5.8	0.187	0.348	0.500	0.205	21.2	42.7
D	0.831	0.427	43.1	−73.1	0.185	0.263	0.143	0.150	72.8	90.0
Leg66	1.117	0.454	47.3	−69.6	0.185	0.263	0.143	0.150	68.7	89.8
Leg4	1.292	1.181	51.2	−95.0	0.207	0.371	0.143	0.234	57.0	63.0

Designs with higher *payload* and *desired velocity* scores (designs B and D) tend to have smaller bases and the minimum lengths required for their legs. This is to reduce the overall weight of the robot allowing more of the force created by their joint torques to be utilized on extra payload. Another interesting trend in these designs is that the last bend angle *α*_2_ tends to be around 90°. This creates a smaller moment arm for the second joint of each of the legs when applying a downwards force on the ground. Therefore, this joint can convert more of its torque into a downward force rather than having to overcome a bigger moment arm as is the case when this bend angle is smaller. Finally, these designs tend to utilize the inherent stiffness of the joints to provide extra torque. Since the foot is located below the walking region at their zero configurations the joints want to spring back when the foot is in the walking region. B and D exhibit this phenomenon. The joint stiffness provides extra torque to lift the robot in addition to the torque provided by the pressure in the joints’ bellows. However, this also appears to be the reason these designs have a lower dexterity score, as they have a smaller quantity of configurations within the walking region.

The more stable robots (designs A and C) tend to have larger bases. A larger base helps in stability since it causes more of the footholds to be further out from the center of gravity and creates a larger support polygon (see Figure 5). Unsurprisingly, the most stable robot, C, has long legs to find some very stable (in terms of the support polygon criteria) configurations. However, the extra weight caused by a larger base decreases their ability to score well in the *average payload* and *average desired velocity* metrics. While highly stable, designs like C are unable to find many configurations with footholds in the walking region (i.e., its heat map is entirely blue). Therefore we suggest that the *average static stability* metric is best utilized either weighted in an overall objective function or used in a multi-objective function (as we do in this work) as opposed to being utilized in a single objective optimization.

Overall, the optimization provides valuable insight into the relationships between the four objectives as well as the different design characteristics of the quadruped that led to better performance in each metric.

## 5 Hardware Validation Experiments

### 5.1 Experiment Description

While simulation trends discussed in Section 4.3 provide valuable design insights by themselves, we also wanted a fundamental verification that these trends hold–even with unknown modeling errors. We expect most of the modeling error to come from the pneumatic joint torque model, due to unmodeled disturbances like hysteresis or nonlinear joint stiffness. Since the *dexterity in walking region* and *average static stability* metrics are calculated largely with kinematic quantities and masses that are directly measurable, we focus the hardware experiments on the other two metrics which depend more on accurate models of joint torques: *average payload* and *average desired velocity*.

Since building a fully mobile quadruped is also not in the scope of this paper, directly observing the *average desired velocity* metric is challenging. Instead we mounted a single leg to a static testing apparatus and examined the ability of two different optimized solutions to apply a downward force. This facilitates an indirect observation of the *average desired velocity* metric and a direct observation of the *average payload* metric. The two experiments we design are as follows:

1. Experiment 1 was meant to verify that the *average desired velocity* metric is physically meaningful. Taking force measurements across the workspace of a leg indicates if a design is able to apply forces that cause desired accelerations. A step consists of swinging the leg forwards, making contact with the ground, pushing through the middle of the workspace, and breaking contact in the back of the workspace. A design incapable of generating higher forces during the beginning stages of a step would be unable to accelerate easily. Hence we expect to see higher force capability in the front and middle of the workspace than in the back. So we measured the maximum force output in each of these locations. Note that the ‘forward’ direction is defined by ${\vec{u}}_{x}$ in Table 2.
2. Experiment 2 was to verify the competing relationship between *average payload* and *dexterity* seen in Figure 8A. As the dexterity metric of a leg design decreases, we expect its payload capability to increase. To accomplish this, we compare the maximum force output in the middle of the workspace of two different designs: one with a higher dexterity score and one with a higher payload score.

For safety, we limited the working pressure range from 0 to 275,790 Pa (40 psi) which is about half of the joint’s maximum pressure range. We define the maximum force output of a leg as the applied downward force when any actuator is near saturation for more than one second [i.e., the pressure in any joint chamber reaches 13,789 Pa (2 psi) or 262,000 Pa (38 psi)]. We define maximum force output this way because once an actuator saturates the continuum joints have lost control authority and may become unstable or deform in unmodeled ways.

We used a configuration estimator, a joint controller, and a force controller during the experiments. The configuration estimation uses orientation data from HTC Vive virtual reality trackers by applying the general method of estimating joint variables using orientation sensors presented in Hyatt et al. (2019). The joints are controlled with a PI controller acting on the joint angle error signal. The force controller is a PI controller with feedforward control. Each controller is used during different parts of the experiment.

**FIGURE 10:**
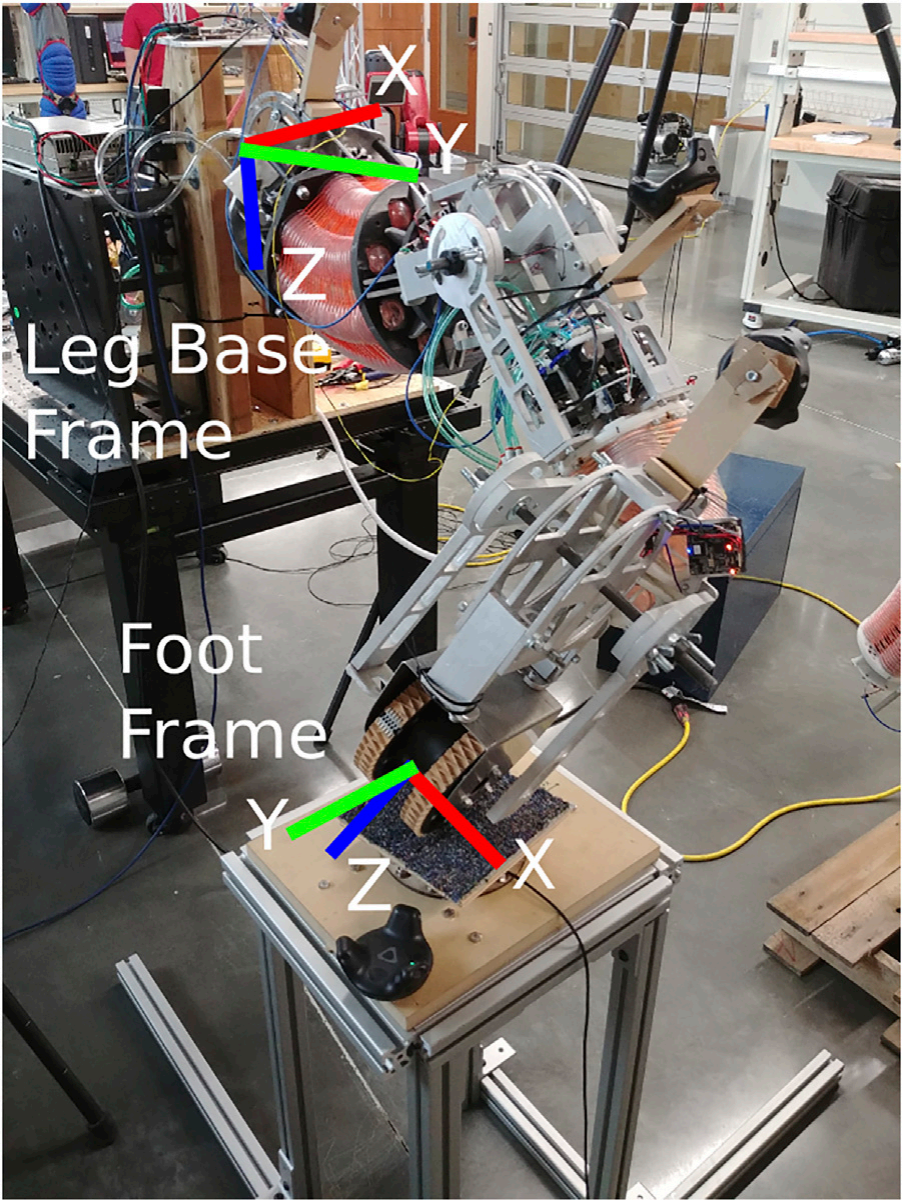
The frame orientations of a constructed leg used in the experiments with the HTC Vive trackers attached to sense its configuration.

Since we model the leg as having four-DoF, the force controller can only actively control forces and torques in four of the six dimensions of a given wrench (generalized vector of forces and torques on a rigid body). Therefore, we command forces in all three directions of the Leg Base Frame and a torque along the *y* axis in the Foot Frame (see Figure 10 for the frames). Controlling a torque about the *y* axis in the Foot Frame helps to mitigate the loss of adequate contact with the ground. To measure the force between the foot and the ground, we use an ATI Mini45 force-torque sensor. This force torque sensor is mounted on top of a rigid stand and beneath a carpeted rectangular platform as shown in Figure 10. During an experiment the leg presses downwards on the carpeted platform while the sensor measures the reaction wrench. The carpet simulates an environment in which the foot may operate and for which we optimized given the estimated coefficient of friction in our lab. However, this could be changed or adapted for different expected friction in different operating environments.

We adhered to the following experimental protocol to measure the maximum force a given leg design can apply:

1. Command the leg to 138 kPa (20 psi) in each bellows to ensure the same starting conditions for all the tests.
2. Command the leg to the joint configuration corresponding to the desired workspace location using the PI joint controller and wait until error is ≤.01 rad for all joint angles.
3. Stop the PI joint-configuration controller, but continue commanding the last pressures from the PI controller to maintain the configuration.
4. Adjust the height the force torque stand until the carpeted platform is barely touching the foot. Verify that the platform is within the walking region *W* as defined in Table 2.
5. Set the current force-torque sensor readings to zero. This ensures that the forces measured at the foot are due to joint torques.
6. Start the force controller. Command zero force in the *x* and *y* axes of the Leg Base Frame and zero torque about the *y* axis in of the Foot Frame. This will keep lateral forces low and maintain good contact with the carpeted platform.
7. Increment the force command along the *z* axis of the Leg Base Frame by 1 N every second until any of the pressure chambers are near saturation for longer than one second.

### 5.2 Validation Designs

For Experiment 1, we chose a design (referred to as Leg4) that was closest to the 95th percentile of the *dexterity in walking region* and *average static stability* metrics. For Experiment 2, we choose a design (referred to as Leg66) closest to the 95th percentile in the *average payload* and *average desired velocity* metrics. This design is theoretically capable of applying higher forces than Leg4 but is less stable and less dexterous. For reference, both of these leg designs are labeled in Figure 8B and their design variables are given in Table 4.

### 5.3 Results

Table 5 shows the results for Experiment 1 run on Leg4. Note that we run 5 trials at each workspace location and report summary statistics to demonstrate repeatability. We see that the expected trends over the workspace are indeed validated. The maximum forces are higher near the front and middle of the workspace and drop in magnitude in the back when the foot will lift for another step. This implies that the *average desired velocity* metric used for the optimization produces physically meaningful results. Recall that the optimization did not have the pressure safety limits that we imposed on the hardware, so the actual magnitudes of these results are less important than the relative values across the workspace.

**TABLE 5:** Experimental results from Experiments 1 and 2. Experiment 1 tests across the workspace of Leg4 to observe force capability that can produce a desired velocity during a step. Experiment 2 compares force outputs in the middle of the workspace between 2 different designs to observe differences in payload capability.

	Leg4			Leg66		
	Max	Median	Mean	Max	Median	Mean
Front	306.19 N	301.54 N	298.48 N	-	-	-
Middle	277.10 N	267.61 N	267.98 N	367.25 N	349.38 N	351.64 N
Back	173.85 N	160.79 N	152.75 N	-	-	-

Table 5 contains the results of Experiment 2. From Figure 8B, we expect that Leg66 will be capable of higher force outputs because of its higher *average payload* score compared to Leg4. This is exactly what we observe. On average, Leg66 is capable of applying downward forces 31.22% higher than Leg4.

While these results are preliminary, they clearly demonstrate the fact that the design metrics presented in this work are capable of mathematically capturing complex characteristics that are desirable for quadrupedal locomotion with compliant continuum joints. They succinctly represent real-world behaviors and capabilities despite modeling inaccuracies and various approximations that we used to preserve numerical tractability.

## 6 Conclusion

We presented four design metrics for the design optimization of any walking robot. We also derived and validated approximations of these metrics, without which a useful optimization would be numerically infeasible. To validate the metrics, and the approximations, we used the metrics in a 10-dimensional design optimization of a 16 degree-of-freedom quadruped robot.

We solved the design optimization problem using a genetic algorithm, which is well-suited to multi-objective optimization problems where understanding design trade-offs via analysis of a Pareto Frontier is valuable. We also included a discussion of these trade-offs for our soft robot quadruped application.

Finally, we built two different designs selected from the optimization results and provided preliminary evidence that the relationships between design variables predicted by the optimization also occur in real-world experiments.

Future work could include comprehensive hardware tests to explicitly compare full functionality of different walking robot designs. This would require the construction of 3 additional legs and a body which could be adjustable along each of the 10 design variables to facilitate quick iteration through different designs. Accomplishing the more general goal of unstructured terrain traversal will also require the development of new large-scale soft robot gait controllers, force controllers, and path planning algorithms tailored to a pneumatically-actuated compliant quadruped. The results presented in this paper show that leg designs optimized for specific metrics are indeed more performant in those metrics. For this reason, we expect that designing a full quadruped robot with these metrics and trade-offs in mind will accelerate the development of robots and controllers towards these general locomotion goals. This expectation is supported because of similar work where we have already shown improvements in related soft robot manipulation due to relevant optimization design work [see Bodily (2017)].

## Data Availability

The datasets and analysis scripts for the hardware validation portion of this study can be found on Figshare (https://doi.org/10.6084/m9.figshare.c.5772848.v2).

## Nomenclature

***γ*_max_**: Orientation tolerance from vertical of a foothold
${\hat{S}}_{payload}$: Approximation of *S*_payload_
***μ***: Coefficient of friction
***τ***: Joint torques
***f***: Friction force
***F*_max_**: Maximum payload weight that a robot can carry
***F*_min_**: A possible value of *f*
***F*_payload_**: Payload capability of a robot design in any given configuration. Defined in Eq. 4
***F*_robot_**: Weight of an entire robot
***g***: Gravitational constant
***J*_*X*,*i*_**: Manipulator jacobian
***m***: Mass
***N***: Average load a single leg is required to bear
***n*_bin,*j*_**: Number of times a leg reaches the *j*th bin
***n*_foot_**: All possible foothold combinations for all legs, used as dexterity in walking region metric for an entire quadruped
***n*_*W*,*i*_**: Number of unique and reachable foot pose bins in *W* for leg *i*, used as the dexterity in walking regions metric for a single leg
***R*_body_**: Reaction force from body at Point A in Figure 1B
***s***: Scaling factor on *N*. See Eq. 6
***s*_*C*_**: General symbol for the portion of a given metric that depends on the configuration of the leg
***s*_approx_**: General symbol for the approximated value of a given metric
***s*_bin,*j*_**: Total score for an individual bin, i.e. combination of all *s*_*C*_ that reaches the *j*th bin
***s*_comb_**: General symbol for the portion of a given metric that depends on the combinations of all the footholds
***S*_payload_**: Payload score accounting for kinematic singularities. Used as the average payload in walking regions metric.
***W***: Task space of a leg
***W*_min_** *F*_min_: represented as a wrench

## References

[B1] Balling R. Wilson S. (2001). “The Maximin Fitness Function for Multi-Objective Evolutionary Computation: Application to City Planning,” in Proceedings of the 3rd Annual Conference on Genetic and Evolutionary Computation (San Francisco, CA: Morgan Kaufmann Publishers Inc.), 1079–1084.

[B2] Birglen L. Ruella C. (2014). Analysis and Optimization of One-Degree of Freedom Robotic Legs. ASME J. Mech. Robotics 6. 10.1115/1.4027234

[B3] Bodily D. M. (2017). Design Optimization and Motion Planning for Pneumatically-Actuated Manipulators. Provo Utah: Brigham Young University.

[B4] Cao J. Qin L. Liu J. Ren Q. Foo C. C. Wang H. (2018). Untethered Soft Robot Capable of Stable Locomotion Using Soft Electrostatic Actuators. Extreme Mech. Lett. 21, 9–16. 10.1016/j.eml.2018.02.004

[B5] Chadwick M. Kolvenbach H. Dubois F. Lau H. F. Hutter M. (2020). Vitruvio: An Open-Source Leg Design Optimization Toolbox for Walking Robots. IEEE Robot. Autom. Lett. 5, 6318–6325. 10.1109/lra.2020.3013913

[B6] Chen S. Cao Y. Sarparast M. Yuan H. Dong L. Tan X. (2020). Soft Crawling Robots: Design, Actuation, and Locomotion. Adv. Mat. Technol. 5, 1900837. 10.1002/admt.201900837

[B7] Cianchetti M. Calisti M. Margheri L. Kuba M. Laschi C. (2015). Bioinspired Locomotion and Grasping in Water: the Soft Eight-Arm OCTOPUS Robot. Bioinspir. Biomim. 10, 035003. 10.1088/1748-3190/10/3/035003 25970014

[B8] Crespi A. Karakasiliotis K. Guignard A. Ijspeert A. J. (2013). Salamandra Robotica II: An Amphibious Robot to Study Salamander-like Swimming and Walking Gaits. IEEE Trans. Robot. 29, 308–320. 10.1109/TRO.2012.2234311

[B9] De Santos P. G. Garcia E. Estremera J. (2007). Quadrupedal Locomotion: An Introduction to the Control of Four-Legged Robots. Springer London: Springer Science and Business Media.

[B10] De Vincenti F. Kang D. Coros S. (2021). “Control-aware Design Optimization for Bio-Inspired Quadruped Robots,” in 2021 IEEE/RSJ International Conference on Intelligent Robots and Systems (IROS) (IEEE), 1354–1361. 10.1109/iros51168.2021.9636415

[B11] Fedorov D. Birglen L. (2015). “Analysis and Design of a Two Degree of Freedom Hoeckens-Pantograph Leg Mechanism,” in ASME 2015 International Design Engineering Technical Conferences and Computers and Information in Engineering Conference (Boston, Massachusetts: ASME), 1–9. 10.1115/detc2015-47330

[B12] Florez J. M. Shih B. Bai Y. Paik J. K. (2014). “Soft Pneumatic Actuators for Legged Locomotion,” in 2014 IEEE International Conference on Robotics and Biomimetics (ROBIO 2014) (IEEE), 27–34. 10.1109/robio.2014.7090302

[B13] Godage I. S. Nanayakkara T. Caldwell D. G. (2012). “Locomotion with Continuum Limbs,” in IEEE International Conference on Intelligent Robots and Systems (IEEE), 293–298. 10.1109/IROS.2012.6385810

[B14] Gülhan M. M. Erbatur K. (2018). Kinematic Arrangement Optimization of a Quadruped Robot with Genetic Algorithms. Meas. Control 51, 406–416. 10.1177/0020294018795640

[B15] Ha S. Coros S. Alspach A. Kim J. Yamane K. (2018). Computational Co-optimization of Design Parameters and Motion Trajectories for Robotic Systems. Int. J. Robotics Res. 37, 1521–1536. 10.1177/0278364918771172

[B16] Hyatt P. Johnson C. C. Killpack M. D. (2020). Model Reference Predictive Adaptive Control for Large-Scale Soft Robots. Front. Robotics AI 7, 558027. 10.3389/frobt.2020.558027 PMC7806097 33501321

[B17] Hyatt P. Kraus D. Sherrod V. Rupert L. Day N. Killpack M. D. (2019). Configuration Estimation for Accurate Position Control of Large-Scale Soft Robots. IEEE/ASME Trans. Mechatron. 24, 88–99. 10.1109/tmech.2018.2878228

[B18] Jin H. Dong E. Alici G. Mao S. Min X. Liu C. (2016). A Starfish Robot Based on Soft and Smart Modular Structure (SMS) Actuated by SMA Wires. Bioinspir. Biomim. 11, 056012. 10.1088/1748-3190/11/5/056012 27609700

[B19] Kim Y. Lee Y. Cha Y. (2021). Origami Pump Actuator Based Pneumatic Quadruped Robot (Oparo). IEEE Access 9, 41010–41018. 10.1109/access.2021.3065402

[B20] Loc V.-G. Koo I. M. Trong T. D. Kim H. M. Moon H. Park S. (2010). “A Study on Traversability of Quadruped Robot in Rough Terrain,” in 2010 International Conference on Control Automation and Systems (ICCAS) (Gyeonggi-do, Korea (South): IEEE), 1707–1711. 10.1109/iccas.2010.5669775

[B21] McGhee R. B. Frank A. A. (1968). On the Stability Properties of Quadruped Creeping Gaits. Math. Biosci. 3, 331–351. 10.1016/0025-5564(68)90090-4

[B22] Mcghee R. B. Iswandhi G. I. (1979). Adaptive Locomotion of a Multilegged Robot over Rough Terrain. IEEE Trans. Syst. Man. Cybern. 9, 176–182. 10.1109/tsmc.1979.4310180

[B23] Morzadec T. Marcha D. Duriez C. (2019). “Toward Shape Optimization of Soft Robots,” in 2019 2nd IEEE International Conference on Soft Robotics (RoboSoft) (Seoul, Korea (South): IEEE), 521–526. 10.1109/robosoft.2019.8722822

[B24] Onal C. D. Rus D. (2013). Autonomous Undulatory Serpentine Locomotion Utilizing Body Dynamics of a Fluidic Soft Robot. Bioinspir. Biomim. 8, 026003. 10.1088/1748-3182/8/2/026003 23524383

[B25] Qin L. Liang X. Huang H. Chui C. K. Yeow R. C.-H. Zhu J. (2019). A Versatile Soft Crawling Robot with Rapid Locomotion. Soft Robot. 6, 455–467. 10.1089/soro.2018.0124 30883283

[B26] Roennau A. Heppner G. Nowicki M. Dillmann R. (2014). “Lauron V: A Versatile Six-Legged Walking Robot with Advanced Maneuverability,” in IEEE/ASME International Conference on Advanced Intelligent Mechatronics, AIM (Besacon, France: IEEE), 82–87. 10.1109/AIM.2014.6878051

[B27] Rogóż M. Zeng H. Xuan C. Wiersma D. S. Wasylczyk P. (2016). Light-driven Soft Robot Mimics Caterpillar Locomotion in Natural Scale. Adv. Opt. Mater. 4, 1689–1694.

[B28] Semasinghe C. Taylor D. Rezazadeh S. (2021). “A Unified Optimization Framework and New Set of Performance Metrics for Robot Leg Design,” in 2021 IEEE International Conference on Robotics and Automation (ICRA) (Xi'an, China: IEEE), 4919–4925. 10.1109/icra48506.2021.9561618

[B29] Shepherd R. F. Ilievski F. Choi W. Morin S. A. Stokes A. A. Mazzeo A. D. (2011). Multigait Soft Robot. Proc. Natl. Acad. Sci. U.S.A. 108, 20400–20403. 10.1073/pnas.1116564108 22123978 PMC3251082

[B30] Sherrod V. G. (2019). Design Optimization for a Compliant, Continuum-Joint, Quadruped Robot. Provo Utah: Brigham Young University. 10.3389/frobt.2022.860020 PMC9310103 35899074

[B31] Uno K. Valsecchi G. Hutter M. Yoshida K. (2021). “Simulation-based Climbing Capability Analysis for Quadrupedal Robots,” in Climbing and Walking Robots Conference (Cham: Springer), 179–191. 10.1007/978-3-030-86294-7_16

[B32] Vikas V. Cohen E. Grassi R. Sozer C. Trimmer B. (2016). Design and Locomotion Control of a Soft Robot Using Friction Manipulation and Motor-Tendon Actuation. IEEE Trans. Robot. 32, 949–959. 10.1109/tro.2016.2588888

[B33] Wang W. Lee J.-Y. Rodrigue H. Song S.-H. Chu W.-S. Ahn S.-H. (2014). Locomotion of Inchworm-Inspired Robot Made of Smart Soft Composite (Ssc). Bioinspir. Biomim. 9, 046006. 10.1088/1748-3182/9/4/046006 25289658

[B34] Zhang J. Liu Q. Zhou J. Song A. (2021). Crab-inspired Compliant Leg Design Method for Adaptive Locomotion of a Multi-Legged Robot. Bristol, England: Bioinspiration and Biomimetics (HQ of IOP publishers, the parent company). 10.1088/1748-3190/ac45e6 34937001

[B35] Zhang S. Liu M. Yin Y. Rong X. Li Y. Hua Z. (2019). Static Gait Planning Method for Quadruped Robot Walking on Unknown Rough Terrain. IEEE Access 7, 177651–177660. 10.1109/access.2019.2958320

[B36] Zou J. Lin Y. Ji C. Yang H. (2018). A Reconfigurable Omnidirectional Soft Robot Based on Caterpillar Locomotion. Soft Robot. 5, 164–174. 10.1089/soro.2017.0008 29297768

